# Metabolic engineering for the production of acetoin and 2,3-butanediol at elevated temperature in *Parageobacillus thermoglucosidasius* NCIMB 11955

**DOI:** 10.3389/fbioe.2023.1191079

**Published:** 2023-05-02

**Authors:** Lili Sheng, Abubakar Madika, Matthew S. H. Lau, Ying Zhang, Nigel P. Minton

**Affiliations:** ^1^ Clostridia Research Group, BBSRC/EPSRC Synthetic Biology Research Centre (SBRC), School of Life Sciences, Biodiscovery Institute, The University of Nottingham, Nottingham, United Kingdom; ^2^ Department of Microbiology, Faculty of Life Sciences, Ahmadu Bello University, Zaria, Nigeria; ^3^ NIHR Nottingham Biomedical Research Centre, Nottingham University Hospitals NHS Trust, The University of Nottingham, Nottingham, United Kingdom

**Keywords:** *P. thermoglucosidasius* NCIMB 11955, acetoin, 2,3-butanediol, thermophile, CRISPR CAS

## Abstract

The current climate crisis has emphasised the need to achieve global net-zero by 2050, with countries being urged to set considerable emission reduction targets by 2030. Exploitation of a fermentative process that uses a thermophilic chassis can represent a way to manufacture chemicals and fuels through more environmentally friendly routes with a net reduction in greenhouse gas emissions. In this study, the industrially relevant thermophile *Parageobacillus thermoglucosidasius* NCIMB 11955 was engineered to produce 3-hydroxybutanone (acetoin) and 2,3-butanediol (2,3-BDO), organic compounds with commercial applications. Using heterologous acetolactate synthase (ALS) and acetolactate decarboxylase (ALD) enzymes, a functional 2,3-BDO biosynthetic pathway was constructed. The formation of by-products was minimized by the deletion of competing pathways surrounding the pyruvate node. Redox imbalance was addressed through autonomous overexpression of the butanediol dehydrogenase and by investigating appropriate aeration levels. Through this, we were able to produce 2,3-BDO as the predominant fermentation metabolite, with up to 6.6 g/L 2,3-BDO (0.33 g/g glucose) representing 66% of the theoretical maximum at 50°C. In addition, the identification and subsequent deletion of a previously unreported thermophilic acetoin degradation gene (*acoB1)* resulted in enhanced acetoin production under aerobic conditions, producing 7.6 g/L (0.38 g/g glucose) representing 78% of the theoretical maximum. Furthermore, through the generation of a Δ*acoB1* mutant and by testing the effect of glucose concentration on 2,3-BDO production, we were able to produce 15.6 g/L of 2,3-BDO in media supplemented with 5% glucose, the highest titre of 2,3-BDO produced in *Parageobacillus* and *Geobacillus* species to date.

## Highlights

• Acetoin and 2,3-butanediol production engineered in a thermophilic Gram-positive chassis.
• Formation of by-products was minimized by deleting competing pathways.
• Identification and subsequent deletion of a previously unreported thermophilic acetoin degradation gene.
• Acetoin produced at 7.6 g/L (0.38 g/g glucose), 78% of the theoretical maximum.
• 2,3-butanediol was the predominant fermentation metabolite, up to 6.6 g/L (0.33 g/g glucose), 65% of the theoretical maximum.

## 1 Introduction

2,3-butanediol (2,3-BDO) and 3-hydroxybutanone (acetoin) are important chemicals with numerous applications in a number of different sectors. 2,3-BDO and its derivatives are involved in the manufacture of food additives and flavorings, pharmaceuticals, printing inks, synthetic rubber and plastics, fuel additives and antifreeze agents in addition to many more (Biswas et al., 2012; Cho et al., 2015; Maina et al., 2022). Its global market value is predicted to be $300 million by 2030 (Chemicals and Materials Market Research Report, 2019). Likewise, acetoin serves as a building block for the synthesis of cosmetics and pharmaceuticals and is also widely used as an additive in food flavoring and electronic cigarettes (Bao et al., 2014; Allen et al., 2016; Kandasamy et al., 2016). In recognition of its versatility, acetoin was named as one of the 30 platform chemicals produced from biomass by the United States Department of Energy (Werpy and Petersen, 2004).

Currently, both chemicals are primarily derived from petrochemicals by chemical-synthesis. However, concerns over the finite nature of fossil fuels and the current climate crisis, coupled with increasing demands, have provided a greater incentive to produce 2,3-BDO and acetoin via sustainable and renewable approaches. Microbial-based fermentations using lignocellulose, a renewable feedstock, has begun to attracted considerable attention in recent years (Xiao and Lu, 2014; Yang et al., 2017; Zhang et al., 2019). This is especially true for acetoin, as using fermentation is substantially more favourable in the food industry due to stringent safety regulations (Bao et al., 2014).

Previous efforts directed at producing 2,3-BDO relied upon using bacteria such as *Klebsiella penumoniae*, *Klebsiella oxytoca* and *Paenibacillus polymyxa,* which were known to produce significant amounts (De Mas et al., 1988; Ramachandran and Goma, 1988; Qureshi and Cheryan, 1989; Häßler et al., 2012). Further increases in the productivity of these native producers were sought through genetic modification (Supplementary Table S1), through which yields of up to 150 g L^−1^ were achieved (Song et al., 2019). However, *Klebsiella* species are pathogenic, compromising their use in industrial production (Yang and Zhang, 2018). Acetoin production was primarily achieved using *Bacillus* species. The best reported yield was obtained using *Bacillus amyloliquefaciens* (Luo et al., 2014), whereby 0.37 g g^−1^ glucose was achieved following an adaptive evolution strategy. More recently, thanks to advances in genetic modification tools, 2,3-BDO and acetoin production has been successfully achieved in a range of non-native, mostly mesophilic hosts, including *Saccharomyces cerevisiae*; *Escherichia coli; Corynebacterium* and *Pichia pastoris* (Yang and Zhang, 2018; Zhang et al., 2019) (Supplementary Table S2).

Meanwhile, the use of thermophiles as industrial platforms has been gaining increasing attention due to their many favourable features. These include, lower contamination risks, higher feed conversion rates and reduced cooling costs. They also confer desirable properties on the growth medium, such as reduced viscosity, reduced energy requirements for mixing and increased diffusion rates and substrate solubility (Chang and Yao, 2011; Keller et al., 2014). Temperatures above 50°C also favour consolidated bioprocessing (CBP), a process that allows the cost-effective, simultaneous enzyme-mediated saccharification and fermentation of lignocellulosic biomass into desired products by a single microorganism (Chinn and Mbaneme, 2015).

Thus far, a very limited number of thermophilic organisms have been exploited for the production of 2,3-BDO and acetoin (Table 1). High titre 2,3-BDO/acetoin yields have been documented using *B. licheniformis* strains (Li et al., 2013) with 94% and 84% of theoretical maximum yield (TMY), respectively. Although encouraging, *B. licheniformis* mainly produces optical inactive meso-2,3-BDO and is difficult to genetically manipulate, which impedes further improvements (Li et al., 2017a; Schilling et al., 2020; Zhou et al., 2020). *Parageobacillus* species also have potential for 2,3-BDO and acetoin production under elevated temperatures. Wild isolate *Geobacillus* sp XT15, (Xiao et al., 2012), produced both acetoin and 2,3-BDO, though at a much lower yield (0.22 and 0.12 g/g glucose). More recently up to 7.2 g L^−1^, representing 72% theoretical maximum pure R,R-2,3-BDO production, was achieved in *Parageobacillus thermoglucosidasius* 95A1 (Zhou et al., 2020) using shake-flask fermentation at 55°C.

**TABLE 1:** Comparison of Acetoin and 2,3-butaneidol production by microorganisms at 50°C and above.

Organism	Genotype and modifications	Fermentation condition	Yield^a^	Source
2,3-Butanediol				
*B. licheniformis*	Strain 10-1-A	Fed-batch, 50°C, pH 7.0	0.47	Li et al. (2013)
*Geobacillus sp*	Strain XT15	Shake flask, 55°C, pH 7.0	0.22	Xiao et al. (2012)
*P. thermoglucosidasius*	Strain DSM 2542: *Δldh/alsSBs-alsDSt*	Shake flask, 52°C, pH 7.0	0.36	Zhou et al. (2020)
*P. thermoglucosidasius*	Strain NCIMB11955:*ΔldhΔpflΔg3pdh1 Δgdh1Δldh2ΔadhEΔack alsSBs-alaDBc*	Falcon tube, 50°C, pH 7.0	0.33	This study
Acetoin				
*B. licheniformis*	Strain WX-02: *ΔbudCΔacoR1*	Shake Flask, 50°C, pH 6.0	0.44	Li et al. (2017b)
*Geobacillus sp*	Strain XT15	Shake Flask, 55°C, pH 7.0	0.12	Xiao et al. (2012)
*P. thermoglucosidasius*	Strain NCIMB11955:*ΔldhΔpflΔg3pdh1 Δgdh1Δldh2ΔadhEΔacoB1alsSBs-alaDBc*	Falcon tube, 50°C, pH 7.0	0.38	This study

^a^ – yield as g per g glucose.

Here, *Parageobacillus thermoglucosidasius* NCIMB 11955 is used as the single chassis to produce both 2,3-BDO and acetoin at elevated temperatures through metabolic engineering. This particular strain is versatile both in term of growth condition and genetic accessibility. It can ferment a wide range of sugars typical of those found in lignocellulosic substrates and has been previously been modified to create an industrially relevant strain, TM242, for ethanol production (Cripps et al., 2009; Hussein et al., 2015). Lately, more sophisticated genetic methods are been developed for the strain, including gene knockout/in using allele coupled exchange (ACE) and CRISPR mediated gene-deletion (Reeve et al., 2016; Sheng et al., 2017; Lau et al., 2021) that together allow for more precise and targeted metabolic engineering. These methods have additionally been used to extend substrate range through the successful expression of the genomically integrated *Caldicellulosiruptor bescii celA* gene enabling direct utilization of lignocellulosic substrate by the organism (Bashir et al., 2019).

To achieve high titre 2,3-BDO and acetoin production, three interrelated approaches were taken by: 1) implementing an efficient acetoin/2,3-BDO pathway through the assembly and expression of genes encoding acetolactate synthase [EC 2.2.1.6] (ALS), acetolactate decarboxylase [EC 4.1.1.5] (ALD) and acetoin reductase/2,3-BDO-dehydrogenase [EC 1.1.1.4] or AR/BDH (Xiao and Lu, 2014); 2) inactivation of genes encoding enzymes participating in competing pathways, and; 3) alleviating redox issues through decoupling of the expression of the native *bdh* using a constitutive promoter and different aeration conditions. The outcome of this strategy were engineered strains that produced 2,3-BDO and acetoin at 50°C at levels close to the theoretical maximum yield (TMY). In both cases, with all major competing pathways disrupted, including ethanol production, up to 90% of the carbon flux entered the acetoin/2,3-BDO pathway, giving scope for further improvement.

## 2 Materials and methods

### 2.1 Chemicals and reagents

All chemicals and reagents were obtained from Sigma-Aldrich Ltd., (Poole, United Kingdom). DNA polymerases, restriction enzymes, T4 ligase and DNA ladders were purchased from New England Biolabs or Promega (Southampton, United Kingdom). EDTA-Free Protease Inhibitor Cocktail Tablets were purchased from Roche (Mannheim, Germany)

### 2.2 Strains and cultivation

All bacterial strains used in this study are listed in Table 2. Unless otherwise stated, *E. coli* Top 10 was used as a host for plasmid construction and *E. coli* B21 (DE3) was used for protein expression. *P. thermoglucosidasius* NCIMB 11955 was used for 2,3-BDO and acetoin production. All *E. coli* strains were grown in Luria-Bertani (LB) agar or LB broth containing appropriate antibiotics (kanamycin: 50 μg/mL; ampicillin: 100 μg/mL) at 30°C or 37°C with agitation of 200 rpm. *P*. *thermoglucosidasius* strains were routinely grown on TSA agar or in 2SPYNG broth (Sheng et al., 2017), with kanamycin when necessary at 12.5 μg/mL, at 50°C–60°C, with agitation of 250 rpm.

**TABLE 2:** List of strains used in this study.

Strains	Genotype	Source
*E. coli* Top10	F-*mcrA ∆ (mrr-hsdRMS-mcrBC) Φ80lacZ∆M15 ∆lacX74 deoR recA1 araD139 ∆ (ara-leu) 7697 galU galK rpsL (StrR) endA1 nupG*	Invitrogen
*E. coli* JM109	*endA1 glnV44 thi-1 relA1 gyrA96 recA1 mcrB* ^+^ Δ(*lac-proAB*) *e14*- [*F′ traD36 proAB* ^+^ *lacI* ^q^ *lacZ*Δ*M15*] *hsdR17*(r_K_^-^m_K_^+^)	Invitrogen
*E. coli* DH5α	F^−^ φ80*lac*ZΔM15 Δ(*lac*ZYA-*arg*F)U169 *rec*A1 *end*A1 *hsd*R17(r_K_^–^, m_K_^+^) *pho*A *sup*E44 λ^–^ *thi*-1 *gyr*A96 *rel*A1	NEB
*E. coli* NEB F^−^	Derivate of DH5α	NEB
*E. coli* BL21(DE3)	F^−^ *omp*T *hsd*S_B_ (r_B_^-^m_B_^-^) *gal dcm rne*131	Promega
*P. thermoglucosidasius*	NCIMB11955 wild type	TMO
LS100	NCIMB11955 Δ*ldh*Δ*pfl*	This study
LS101	LS100-Δ*g3pdh1*	This study
LS102	LS100-Δ*gdh1*	This study
LS103	LS100-Δ*ldh2*	This study
LS104	LS100-Δ*g3pdh1*Δ*gdh*1	This study
LS105	LS100-Δ*g3pdh1*Δ*gdh1*Δ*ldh2*	This study
LS106	LS100-Δ*acoB1*	This study
LS107	LS100-Δ*acoB2*	This study
LS108	LS100-Δ*acuA*	This study
LS109	LS100-Δ*acuB*	This study
LS110	LS100-Δ*acuC*	This study
LS111	LS100-Δ*acuA-C*	This study
LS200	LS100-Δ*adhE*	This study
LS201	LS101-Δ*adhE*	This study
LS203	LS103-Δ*adhE*	This study
LS204	LS104-Δ*adhE*	This study
LS205	LS105-Δ*adhE*	This study
LS300	LS200-Δ*acoB1*	This study
LS301	LS201-Δ*acoB1*	This study
LS303	LS203-Δ*acoB1*	This study
LS304	LS204-Δ*acoB1*	This study
LS305	LS205-Δ*acoB1*	This study
LS400	LS300-Δ*bdh*	This study
LS401	LS301-Δ*bdh*	This study
LS403	LS303-Δ*bdh*	This study
LS404	LS304-Δ*bdh*	This study
LS405	LS305-Δ*bdh*	This study
LS105Δack	LS105-Δ*ack*	This study
LS206	LS205-Δ*ack*	This study
LS306	LS305-Δ*ack*	This study

To assess growth on acetoin and 2,3-BDO, WT *P. thermoglucosidasius* was grown in CBM (Morris, 1971) (consisted of, per litre of deionized water, 200 mg MgSO4·7H_2_O, 7.58 mg MnSO_4_·H2O, 10 mg FeSO_4_·7H_2_O, 1 mg p-aminobenzoic acid, 2 μg biotin, 1 mg thiamine, 4 g acid hydrolysed casein hydrolysate, 0.5 g K_2_HPO_4_ and 0.5 g KH_2_PO_4_) with appropriate carbon sources at 1%.

For acetoin and 2,3-BDO production, typically, appropriate strains from a 10 mL overnight (O/N) preculture were inoculated to prewarmed modified ASYE medium (2 mM MgSO_4_, 0.1 mM CaCl_2,_ 2 mM citric acid, 100 μM FeSO_4_·7H_2_O, 16.85 mM NiCl_3_·6H_2_O, 12.5 mM biotin, 0.1 M HEPES buffer, 0.01 g L^−1^ thiamine, ×5 dilution of Sigma-Aldrich Ltd., M9 minimal salts, ×1,000 dilution of Trace Metal Mix A5, 2% yeast extract, 1%–7% glucose) at a 10% (v/v). Fermentation was carried out on a rotatory shaker at 250 rpm at 50°C for 48 h in sealed 50 mL Falcon tubes with various volumes as indicated before analysis.

### 2.3 General cloning and transformation

Plasmids and primers used in this study are listed in Supplementary Tables S3,S4. All primers were designed using ApE (University of Utah) and analysed with OligoAnalyzer Tool (IdtDNA). For the amplification of the desired DNA products, Phusion High-fidelity DNA polymerase was used for cloning purpose and dream Tag Green PCR (Thermo Fisher Scientific, United Kingdom) master mix was used for strain verification. DNA fragments that had undergone restriction digestions were purified using Zymoclean™ Gel DNA Recovery Kit (ZymoResearch, United Kingdom) before ligation using LigaFast™ Rapid DNA Ligation System (Promega). Chemically competent *E. coli* Top10 cells produced in-house were used to propagate the constructed plasmids and plasmids were harvested using Monarch^®^ Plasmid Miniprep Kit (New England BioLabs). All cloning procedures were conducted following the manufacturer’s instructions. Sanger sequencing (Source Biosciences; EurofinsDNA) were routinely performed to confirm the authenticity of plasmid constructs. Preparation of *P. thermoglucosidasius* electro-competent cells and electroporation procedure using Genepulser electroporator (BioRad, United Kingdom) was undertaken in accordance with the method previously described (Sheng et al., 2017).

### 2.4 Construction of 2,3-BDO biosynthesis pathway

All of the plasmid constructs carrying the genes of the 2,3-BDO biosynthesis pathway are based on pMTL61110 (Sheng et al., 2017), which carries the ColE1 and pUB110 origins of replication, a thermostable kanamycin resistance gene and multiple cloning sites (MCS). Parts included the T1T2 double terminator of the *E. coli rrnB* gene, the P_*ldh*_ promoter of the *Bacillus stearothermophilus ldh* gene and the ALS and ALD genes synthesized by Genewiz Ltd., and codon optimized for *P. thermoglucosidasius* NICMB 11955. Operons were constructed in a stepwise manner. First, P_*ldh*_ was fused with the ALS gene using SOEing PCR and primers N_pLDH_F_AvrII, N_pLDH_BALS_R, N_BALS_LDH_F and N_BALS_R_NdeI, which generated a DNA fragment flanked by AvrII and NdeI restriction sites. Following successful ligation into LS-ALS1, the *P. thermoglucosidasius* GPAD gene promoter (P_*gpad*_) was fused with the ALD(n) gene concomitant with the introduction of a synthetic RBS (ribosome binding site). Primers used were GPAD_F_NotI, GPAD_R_Bot9, ALD(n)_Bot9_F and ALD(n)_R_XhoI. The resultant DNA fragment, flanked by NotI and XhoI restriction sites, was combined with the previous fragment carrying ALS to give plasmid pMTL-BD(n) (where n = 1, 3 and 5.) Plasmid pMTL-BD3b could not be propagated in *E. coli* due to cell toxicity, instead, inserts containing the promoter of the *rplS* gene fused to the native *bdh* gene (SOEing PCR with primers BDH1_F_PheB, BDH1_R_NheI, Rpls_F_XhoI, Rpls_R_PheB) were ligated with pMTL-BD3 after restriction digest with XhoI and NheI and electroporated directly into NICMB 11955 following dialysis treatment. Colony PCR and Sanger sequencing were used to verify that each plasmid was correct.

### 2.5 Strain construction using CRISPR

All constructs for various targeted gene knockouts were based on pMTL67555 (Lau et al., 2021) which is based on the *Streptococcus thermophilus* Cas9-3. Synthetic knockout cassettes contained target specific sgRNA, identified using Benchling CRISPR Guide Design software (www.benchling.com), and homology arms (HA) synthesized by Genewiz Ltd. HA consists of 501 bp regions of DNA from up- (5′) and down- (3′) stream of the gene to be inactivated. Ligation of the fragments between the BamHI and AscI restriction sites of pMTL67555 yielded the knockout plasmids. Deletion cassettes were designed to generated an in-frame deletion of the targeted gene comprising just 3 codons, including the start and stop codons. Following the successful transformation of the knockout plasmids into NICMB 11955, transformants are passaged on TSA-kanamycin agar for 3 days before verification of gene knockout by colony PCR. Once verified, the mutants were passaged for at least 6 times in 2SPYNG without selection (at 12 h intervals) for plasmid curing.

### 2.6 Enzyme assays

Crude cell extracts were prepared using BugBuster 10X protein extraction reagent (Merck). The protein extraction buffer (1X) consisted of BugBuster 10X (2 mL), sodium phosphate buffer 50 mM (18 mL), EDTA-free protease inhibitor tablet (Roche), and lysozyme (20 mg/mL). Total protein concentrations of the soluble fraction of cell lysates were determined using BCA-kit (Thermo Fisher Scientific, United Kingdom) following the manufacturer’s instructions and a standard curve generated from BSA (200–1,000 μg/mL) was used to calculate the protein concentrations. Assays for acetoin reductase activity were carried out at 60°C, initiated by adding 10 μL crude cell lysate to a solution containing 50 mM acetoin, 0.5 mM NADH or NADPH and 1 mM TCEP (Nicholson, 2008). Oxidation of NADH to NAD^+^ was monitored spectrophotometrically by the AR dependent rate of A_340_ decrease. Acetolactate decarboxylase activity was assayed as previously described (Dulieu and Poncelet, 1999). In short, substrate solution was prepared by diluting 0.05 mL of ethyl 2-acetoxy-2-methylacetoacetate with 1.5 mL of H_2_O and 1.5 mL of 1 M NaOH. After stirring for 20 min, it was made up to 25 mL with 50 mM MES, adjusting to pH 6.0 using 0.5 M HCL. The reaction was initiated following incubation of cell lysates with substrate at 60°C for 20 min before adding Voges-Proskauer Reagent A and Reagent B and 280 µL 0.5% (w/v) creatinine. After incubation at 40 min at room temperature, A_522_ was used for quantification of the final acetoin concentration using an acetoin standard curve. Negative controls included reaction with and without substrate in the absence of ALD. One Unit of ALD activity is defined as the concentration of acetoin by ALD (absorbance/a constant molar coefficient ε) per 1 mg of protein.

### 2.7 Analytical methods

Growth of all bacterial cultures was monitored by measuring optical density at 600 nm (OD_600_) using a Pharmacia Novaspec II. Metabolites were analysed using high-performance liquid chromatography (HPLC-UV and RI), performed using the Dionex UltiMate 3,000 System and 5 mM H_2_SO_4_ as the mobile phase. Valeric acid at 50 mM was used as an internal standard. The sample supernatants were filtered with 0.2 μm filters and mixed in 1:1 ratio with diluent. The Bio-Rad Aminex HPX-87H 300 mm × 7.8 mm × 9 μm column was used for sample elucidation, running at a flow rate of 0.5 mL/min, at 35°C for 55 min. All experiments were performed in triplicate and data analysed using PRISM (GraphPad Software, La Jolla, United States) and Excel (Microsoft).

## 3 Results

### 3.1 Verification of the native acetoin/2,3-BDO pathway

The route to 2,3-BDO from pyruvate involves the intermediate metabolites acetolactate and acetoin, catalyzed by ALS, ALD and BDH. The *P. thermoglucosidasius* NICMB 11955 genome possesses genes encoding homologues of ALS (BCV53_05415) and BDH (BCV53_03685) but not ALD. Although it may still be able to produce 2,3-BDO via the oxygen-dependent, spontaneous oxidation of acetolactate to di-acetyl, no trace was evident in culture supernatants following growth of the WT in ASYE medium. The major metabolites (Supplementary Figure S1) were lactate (74%), acetate (16%) and ethanol (8%).

In previous work (Sheng et al., 2017), various *P. thermoglucosidasius* mutants were constructed by allelic exchange using a heterologous *pyrE* gene as a counter selection marker and the requisite Δ*pyrE* derivative of NICMB 11955. These included a Δ*ldh*Δ*pfl* double mutant in which the major fermentative pathways to lactate and formate had been eliminated. In such mutants, pyruvate is accumulated to high (37 mM) concentrations (Extance et al., 2013). We speculated that the acetyl-CoA pathway in this mutant would be overloaded and carbon flux would overflow into the acetoin/2,3-BDO pathway. To test this hypothesis, we first repaired the *pyrE* mutant allele to wildtype using ACE. The strain generated, LS100, was shown to produce up to 5 mM 2,3-BDO under the same conditions as when the WT produced none (Supplementary Table S5).

The data obtained with LS100 provided indirect evidence that the *bdh* gene identified in the *P. thermoglucosidasius* NICMB 11955 genome encodes a BDH with activity against acetoin. To confirm this, we PCR amplified, cloned and expressed the gene in *E. coli* using the PET expression system (pET17b). Enzyme assays with acetoin as the substrate were conducted at 60°C using crude cell extracts of the *E. coli* clone generated. These showed (Supplementary Figure S2) that the enzyme is an NADH dependent alcohol dehydrogenase with significant activity towards acetoin at thermophilic temperatures.

### 3.2 Selection and characterization of pathway components

The native *P. thermoglucosidasius* ALS is not an ideal candidate for acetolactate production as it is anabolic, belonging to the branched chain amino acid biosynthetic pathway. Enzymes of this type are generally highly regulated by product inhibition (Weinstock et al., 1992; Elisáková et al., 2005). Essential to acetolactate formation is a catabolic ALS not subject to product inhibition*.* Previous work indicates that the *Bacillus subtilis* ALS (ALS1) is a suitable candidate (Lin et al., 2014; Zhou et al., 2020). Interestingly, we found that ALS1 shares near identical (99%) AA sequence homology to the ALS of *B. licheniformis*. In addition, we also identified another ALS, from *Bacillus coagulans* 2–6 (ALS2) (Wang et al., 2014), a species shown to produce acetoin and 2,3-BDO as the primary fermentation products under aerobic conditions (Su and Xu, 2014). ALS2 is annotated as being catabolic and shares 65% AA homology with ALS1.

The conversion of acetolactate to acetoin also needs to be efficiently catalyzed. An effective ALD is, therefore, essential. Furthermore, it is desirable to have a wide selection of pathway components to give greater flexibility for a more integrated approach to metabolic engineering, as sometimes the introduction of high enzyme activities throughout a pathway, especially in heterologous hosts, can lead to depletion of intermediates, causing diminished growth, cell toxicity, and reduced product yields (Lechner et al., 2016).

Genome sequences of the two thermophilic producers (Xiao et al., 2012; Li et al., 2017b) were unavailable and reports of thermophilic ALDs are rare. Hence, six novel ALDs, from a wide range of organisms able to grow at 50°C and above, were selected as potential candidates. The only mesophile source in this selection was the ALD of *Exiguobacterium acetylicum* (formerly *Brevibacterium acetylicum*). However, previous reports have indicated that its ALD (ALD1) possessed promising properties including stability at 60°C, high activity at 50°C and a broad pH range of 5–8 (Ohshiro et al., 1989). Others include two from bacilli, ALD2 (*B. coagulans*) (Wang et al., 2014) and ALD3 (*Bacillus cereus*) (Finlay et al., 2000); one from an anaerobe, ALD4 (*P. thermopropionicum*) (Imachi et al., 2002) and two fungal ALDs, ALD5 (*Chaetomium thermophilum*) (Kellner et al., 2016) and ALD6 (*Thielavia terrestris*) (Berka et al., 2011).

**FIGURE 1:**
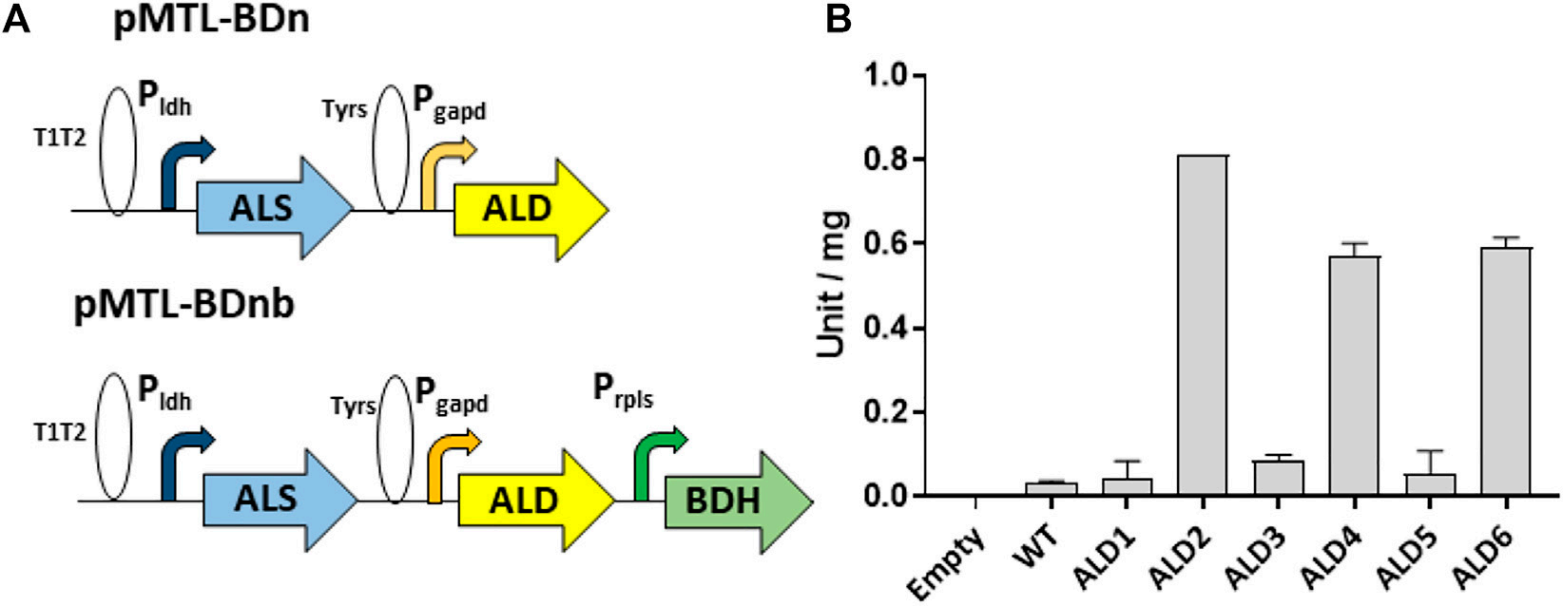
Assembly of enzyme variants in the acetoin/2,3-BDO pathway and assay of acetolactate decarboxylase activities. **(A)** Gene configuration of the constructed acetoin/2,3-BDO pathway. The ALS gene is expressed from the *G. stereothermophilus* lactate dehydrogenase promoter (P_*ldh*_) with the native RBS, the ALD gene is expressed via PT glyceraldehyde 3-phosphate dehydrogenase promoter and RBS9 (TAG​ATA​AAG​GAG​GTA​GAA​CT, taken from the RBS of the locus tag CLB_1812 of *Clostridium botulinum* ATCC 3502, accession no. CP000726) while the BDH gene is expressed from the native *rplS* promoter and *G. stereothermophilus pheB* RBS. T1T2 and TyrS are transcriptional terminators from the *E. coli rrnB* and *B. subtil*is *tyrS* genes, respectively. **(B)** Assay of α-acetolactate decarboxylases expressed in PT. Y-axis represents unit per mg of protein in the crude cell lysate. Error bars represent standard deviation, n = 3.

The synthetic genes (Genewiz Ltd.) encoding the ALD candidates were codon optimized and cloned into the *P. thermoglucosidasius* expression vector pMTL61110_pldh between its XbaI and KpnI restriction sites. This positioned the ALD genes between the strong P_*ldh*_ promoter of the *G*. *stearothermophilus ldh* (lactate dehydrogenase) gene (Bartosiak-Jentys et al., 2013) and the T1T2 double terminator of the *E. coli rrnB* gene (Figure 1). The 3’ 6 bp spacer sequence of the native P_*ldh*_ RBS were replaced with the palindromic XbaI recognition sequence for convenience of cloning. The plasmids were transformed into WT *P. thermoglucosidasius* NICMB 11955 and the ALDs were allowed to express before crude cell lysates were prepared and assayed for acetolactate dehydrogenase activity (Materials and Methods). Significant activities were observed in extracts made from cells producing three of the ALDs. ALD2 had the highest activity (0.81 ± 0.00 U/mg), followed by ALD6 and ALD4, which exhibited 73% and 70%, respectively, of the activity of ALD1 (Figure 1).

### 3.3 Implementation of acetoin/2,3-BDO pathway in *P. thermoglucosidasius*

The components of the acetoin/2,3-BDO operons were assembled such that the ALS and ALD genes were placed under the control of the P_*ldh*_ and P_*gapd*_ promoters, respectively (Figure 1). To prevent the possibility of recombination between identical sequences, the native *ldh* RBS preceded ALS genes, while ALD genes relied on the Bot9 RBS (Lau et al., 2021). Each promoter-gene cassette was separated by two unique restriction sites (SbfI/KpnI for ALS and NotI/XhoI for ALD) for the convenience of replacing/swapping cassettes.

During the assembly of the various combinations of ALS and ALD genes, a major obstacle observed was cell toxicity both when cloning, in *E. coli* and when the shuttle vectors were transferred into the *P. thermoglucosidasius* host. Four *E. coli* strains (Top10, JM109, DH5α and NEB F^−^) were tested and only JM109 and NEB F^−^ allowed propagation of the desired plasmids. No growth was evident in the case of DH5α, while although Top10 transformants grew, the plasmids were found to have acquired mutations effecting the integrity of the cloned genes. Insertions/deletions and nucleotide substitutions that respectively caused frameshifts and nonsense mutations in the ALD gene proved to be a frequent occurrence when plasmids were introduced into *P. thermoglucosidasius.* In total, just three cassettes (BD2, BD3 and BD5) were obtained that were cloneable in *E*.*coli* and which maintained structural integrity (verified by Sanger sequencing) for at least three passages following transformation into *P. thermoglucosidasius*. Plasmids carrying these cassettes (encoded enzymes in brackets), were pMTL-BD2 (ALS2-ALD1), pMTL-BD3 (ALS1-ALD3) and pMTL-BD5 (ALS1-ALD5).

**FIGURE 2:**
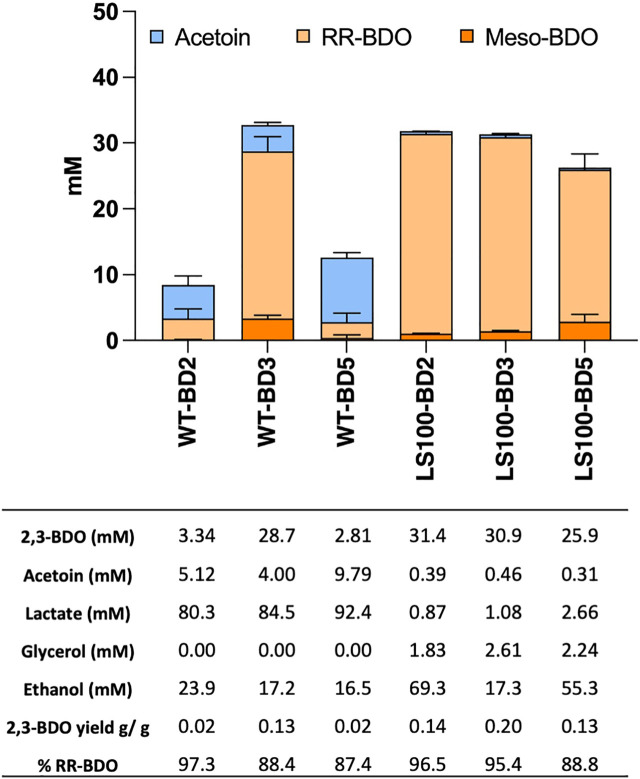
Product profiles of the *P. thermoglucosidasius* WT and LS100 (Δ*ldh*Δ*pfl*) double mutant strain carrying the assembled, plasmid-based, heterologous acetoin/2,3-BDO pathways. Strains carried either plasmids pMTL-BD2, pMTL-BD3 or pMTL-BD5. Cultures were grown for 48 h in 10 mL of M-ASYE medium containing 2% (w/v) yeast extract and 2% (w/v) glucose in 50 mL falcon tubes at 50°C with shaking (250 rpm). Concentrations are calculated as the average three biological replicates. Error bars represent the standard deviation. BDO = 2,3- butanediol, RR-BDO = RR-2,3-butanediol, meso-BDO = meso-2,3- butanediol.

Acetoin and 2,3-BDO levels were characterized in transformants of both the *P. thermoglucosidasius* WT and the LS100 mutant carrying plasmids pMTL-BD2, pMTL-BD3 and pMTL-BD5 to identify which combination of ALS and ALD was the most productive (Figure 2). Levels of 2,3-BDO were increased in all the strains. For the WT, cells harbouring pMTL-BD3 produced up to 29 mM 2,3-BDO, whereas cells carrying pMTL-BD2 and pMTL-BD5 produced much lower levels of 3.34 mM and 2.81 mM, respectively. For LS100, up to 31 mM 2,3-BDO was produced by cells carrying either pMTL-BD2 or pMTL-BD3, with a lower 26 mM for those cells harbouring pMTL-BD5, representing a 5.9-, 6- and 4.8-fold increase, respectively, compared to the same strain without the ALS-ALD operons. For acetoin, a low level (4–10 mM) was evident in all WT strains carrying all three operons whilst insignificant quantities were evident in all LS100 strains. Taking the amount of glucose consumed into account, LS100 cells carrying pMTL-BD3 had the highest 2,3-BDO yield at 0.2 G/G glucose (g/g) This equates to 40% of theoretical maximum yield (TMY). Remarkably, most of the 2,3-BDO produced was the RR-stereoisomer with up to 95% optical purity. This is in contrast to the ratio observed between the meso- and RR-2,3-BDO forms in the plasmid-free LS100 strain which is 1:1. The formation of the different 2,3-BDO isomers is dependent upon acetoin stereoisomerism, where the 3 S form arises through the spontaneous di-acetyl route and the 3R form results from the active ALD route. We concluded that ALD contributed to all of the increase in 2,3-BDO level, and that the native BDH is chiral-specific. This has industrial significance as pure chiral compounds are more valuable and highly sought after (Fu et al., 2016; Kandasamy et al., 2016).

### 3.4 Deletion of competing pathways

The Embden-Meyerhof-Parnas (EMP) pathway requires two NAD + for the production of ATP. In aerobes, NAD+ is recycled by the oxidation of NADH by molecular oxygen. Under oxygen-limiting conditions, a number of fermentative routes in P. thermoglucosidasius are utilized for redox recycling. While they help to maintain redox balance, carbons diverted into various chemicals severely impact the yield of the desired product. A number of studies have shown that deleting genes (*ldh* and *adhE*) of competing pathways (lactate and ethanol formation) are essential for elevated production of 2,3-BDO (Jantama et al., 2015; Radoš et al., 2015; Erian et al., 2018). Although the major fermentative routes to lactate and formate have been deleted in strain LS100, its metabolite profile indicated that lactate and glycerol were still being made as well as significant quantities of ethanol. Accordingly, the inactivation of genes encoding the enzymes involved in their production could be beneficial to 2,3-BDO production.

**FIGURE 3:**
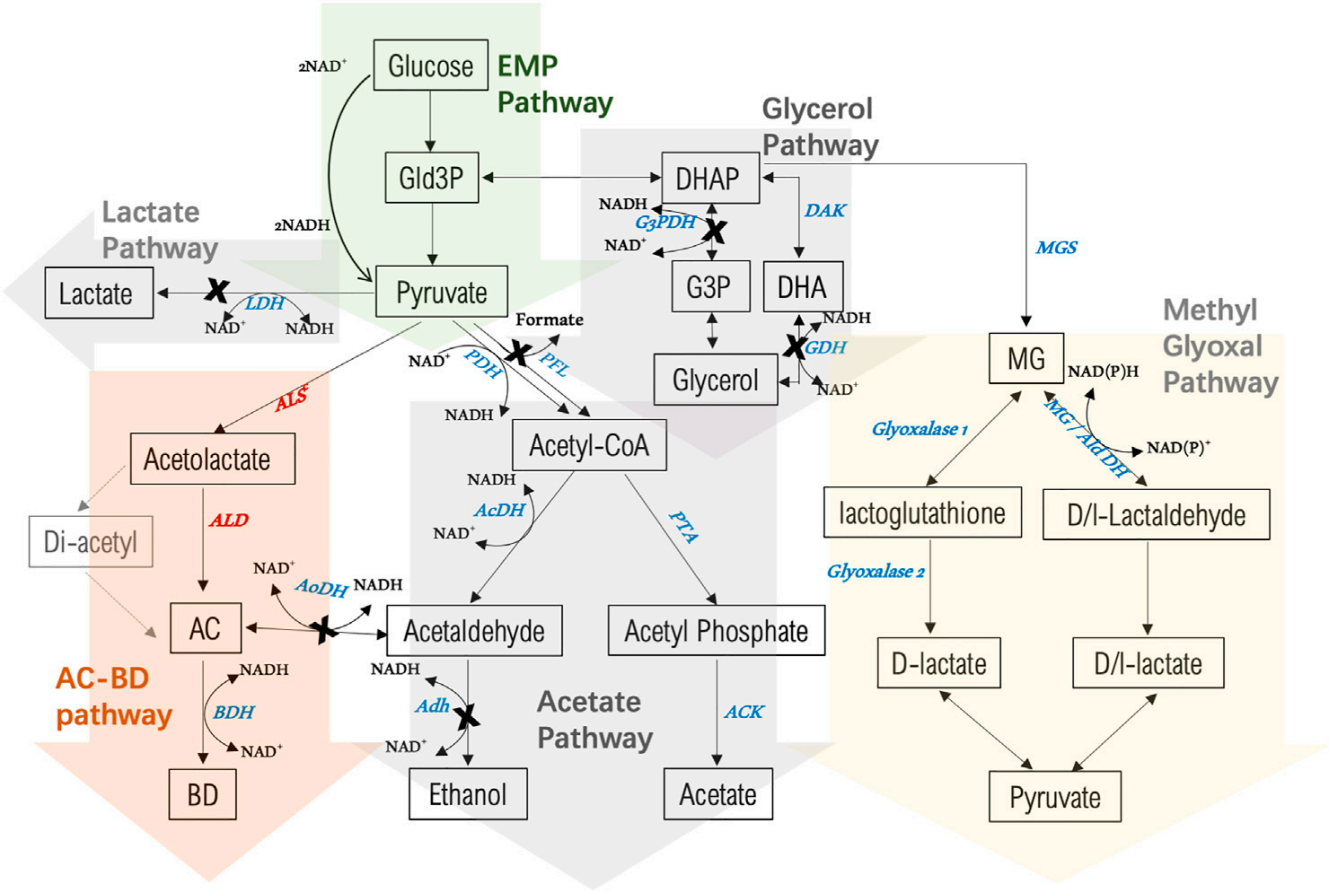
Overview of 2,3-BDO biosynthesis and by-product pathways in *P. thermoglucosidasius* NCIMB 11955 starting from glucose as a substrate. Enzymes involved in 2,3-BDO biosynthesis are in red: ALS: acetolactate synthase; ALD: acetolactate decarboxylase; BDH: 2,3-butanediol dehydrogenase. Enzymes of competing pathways are in blue: LDH: lactate dehydrogenase; G3PDH: glycerol-3-phospahte dehydrogenase; DAK: dihydroxyacetone kinase; GDH: glycerol dehydrogenase; AcDH: Acetaldehdyde dehydrogenase; AoDH: Acetoin dehydrogenase; Adh: alcohol dehydrogenase; PTA: phosphate acetyltransferase; ACK: acetate kinase; MGS: methylglyoxal synthase; AldDH: aldehyde dehydrogenase. A cross indicates where the encoding gene has been deletion in the present study. AC = acetoin, BD = 2,3-butanediol.

Prevention of ethanol formation could be most simply achieved (Figure 3) by knocking out the *adhE* gene (BCV53_03330) as reported previously (Extance et al., 2013). Although the *ldh* gene is already deleted in strain LS100, analysis of the NICMB 11955 genome revealed the presence of another gene encoding a secondary LDH, annotated as L-LDH (BCV53_05985) and sharing no sequence similarities to other non-*P. thermoglucosidasius* strains. The gene was designated *ldh2*. In the case of glycerol, biosynthesis can occur either via glycerol-3-phosphate dehydrogenase (G3PDH) (Albers et al., 1996) or via glycerol dehydrogenase (GDH) (Piattoni et al., 2013; Wang et al., 2016). Two copies of G3PDH exist in *S. cerevisiae*, and deletion of either reduced the production of glycerol while elimination of both entirely blocked glycerol biosynthesis (Medina et al., 2010; Hubmann et al., 2011). When coupled with the 2,3-BDO pathway, increased production was observed (Albers et al., 1996). Two G3PDHs (1,2) (BCV53_15365, BCV53_15685) and two GDHs (1,2) (*gldA*, BCV53_10775) are present in *P. thermoglucosidasius* NICMB 11955. G3PDH1, also annotated as a FAD dependent oxidoreductase, encodes a 550 AA protein sharing 67% and 30% sequence identity to B. *subtilis* and S. *cerevisiae*, respectively. G3PDH2 codes for a 348 AA enzyme that is highly conserved across species (97% in bacilli and 87% in *S. cerevisiae*). GDH1 encodes a 363 AA protein sharing 61% identity to the GDH of *K. pneumoniae* and 50% to that of *B. subtilis* while GDH2 is 358 AA with no significant sequence identity to proteins in any other genus.

Using LS100 as the parental strain, the following deletion mutants were created: LS101 (*ΔldhΔpflΔg3pdh1*); LS102 (*ΔldhΔpflΔgdh1*); LS103 (*ΔldhΔpflΔldh2*), LS104 (*ΔldhΔpflΔg3pdh1Δgdh1*); LS105 (*ΔldhΔpflΔg3pdh1Δgdh1Δldh2*); LS200 (LS100Δ*adhE*); LS201 (LS101Δ*adhE*); LS202 (LS102*ΔadhE*); LS204(LS104Δ*adhE*) and LS205 (LS105Δ*adhE*). Despite numerous attempts, the two genes *g3pdh2* and *gdh2* could not be deleted. Temporary deletions were observed and confirmed by PCR, however, curing the deletion vector proved problematic and when the plasmid was lost reversion back to the parental strain was always observed. It is likely that those are essential genes, for example, the highly conserved *g3pdh2* could be used for the biosynthesis of phospholipid membrane (Coleman et al., 2019).

**FIGURE 4:**
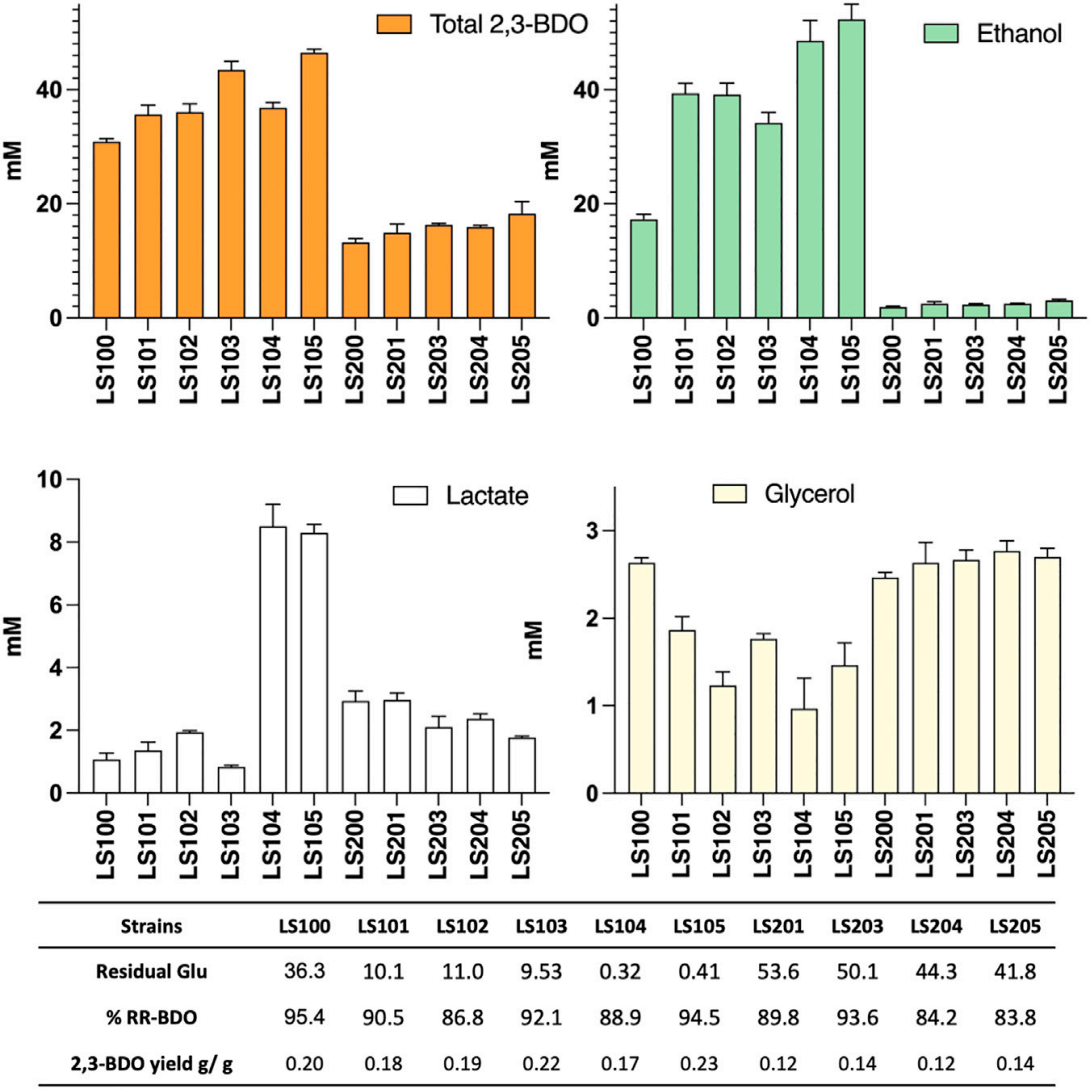
The effect on acetoin and 2,3-BDO yields of targeting various competing pathways in strains with (LS100—LS105) and without (LS200—LS205) a functional *adhE* gene. All strains carry plasmid pMTL-DB3 and were grown at 50°C for 48 h in 10 mL of M-ASYE medium containing 2% (w/v) glucose and yeast extract in a 50 mL falcon tube with shaking (250 rpm). 2,3-BDO level represents the sum of meso-2,3-butanediol and (R,R)-2,3-butanediol. The presence of an *adhE* deletion almost eliminated ethanol production but inhibited growth and 2,3-BDO production. Acetoin is not detected under this condition. Concentrations are calculated as the average of three biological replicates. Error bars represent standard deviation. BDO = 2,3- butanediol, RR-BDO = RR-2,3-butanediol. Strains with genes deleted are indicated as follows: LS100 (*ΔldhΔpfl*), LS101 (*ΔldhΔpflΔg3pdh1*), LS102 (*ΔldhΔpflΔgdh1*), LS103 (*ΔldhΔpflΔldh2*), LS104 (*ΔldhΔpflΔg3pdh1Δgdh1*), LS105 (*ΔldhΔpflΔg3pdh1Δgdh1Δldh2*), LS200 (LS100Δ*adhE*), LS201 (LS101Δ*adhE*); LS202 (LS102*ΔadhE*); LS204(LS104Δ*adhE*) and LS205 (LS105Δ*adhE*).

The various mutant strains obtained were transformed with pMTL-BD3 and the fermentation profiles of the resultant transformants analyzed (Figure 4). Other than the *adhE* knockouts, improvements were observed for all the additional mutants compared to LS100. Mutants carrying Δ*ldh2* had the highest 2,3-BDO level, with 44 mM and 50 mM (0.22 and 0.23 g/g) for LS103 and LS105, respectively. No differences were observed for the single and double glycerol pathway mutants. Ethanol levels were also increased, with up to a 3-fold increase with LS105. For the AdhE mutants, ethanol production was largely eliminated, but 2,3-BDO levels were much reduced to levels of between 13 and 19 mM. Lactate levels increased slightly (1.5–2.0 mM) with single *g3pdh1* deletions and were reduced in the single *ldh*2 mutant (0.8 mM). However, with both *g3pdh1* and *gdh1* deleted, the lactate level increased significantly (up to 9 mM) in strains with or without Δ*ldh*, indicating the presence of alternative routes. Other studies (Loftie-Eaton et al., 2013) suggest that the methylglyoxal pathway most likely contributes to this increase. *P. thermoglucosidasius* NICMB 11955 appears to possess this pathway: methylglyoxal synthase (BCV53_15505), three glyoxylase enzymes (BCV53_06495, BCV53_09900, BVC53_15250) and methylglyoxal reductase (BCV53_06315).

Glycerol levels were reduced in all non-Δ*adhE* strains, with the LS102 and LS104 producing only 1.1 and 0.6 mM, respectively, most likely a consequence of the redox imbalance caused by the loss of the capacity to regenerate NAD+ with the loss of the ethanol pathway. Thus, in the Δ*adhE* strains, both lactate and glycerol levels were significantly increased, despite almost double the amount of residual glucose being left in the media compare to the corresponding non*-adhE* mutants.

### 3.5 Addressing redox constraints in *ΔadhE* mutants

With the disruption of lactate production, the acetyl-CoA pathway plays a major role in maintaining redox balance under oxygen limitation. It follows that ethanol is a major by-product during 2,3-BDO fermentation and deletion of *adhE* reduces growth and productivity. Whereas Zhou *et al.* (2020) reported a decrease in titre and an increase in yield for 2,3-BDO in the ADH mutants in *P. thermoglucosidasius* strain DSM 2542, we observed both a decrease in titre and yield. However, it is still desirable to maximize carbon flux to the acetoin/2,3-BDO pathway necessitating alternative ways of co-factor regeneration.

**FIGURE 5:**
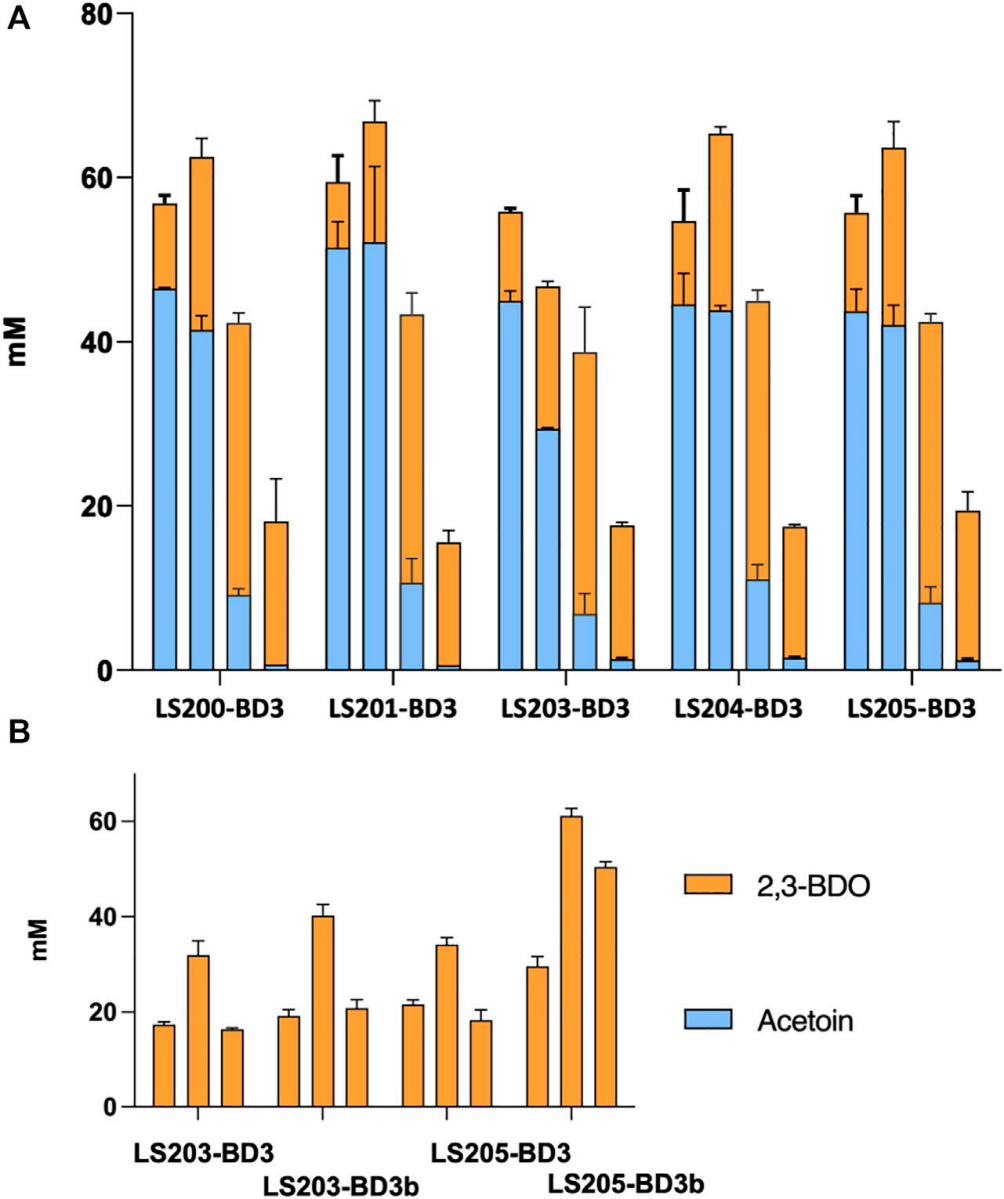
The effect of aeration and BDH overexpression on yields of acetoin and 2,3-BDO. **(A)** Combined acetoin and 2,3-BDO concentrations in Δ*adhE* strains (LS200-LS205) habouring plasmid pMTL-BD3 under different aeration conditions. The media employed was M-ASYE medium containing 2% (w/v) yeast extract and glucose. All fermentations were undertaken at 50°C for 48 h and in 50 mL falcon tubes with shaking (250 rpm). High to low aeration conditions were imposed by the use of either 2 mL, 3 mL, 5 mL or 10 mL culture volumes, represented by column 1–4, left to right. Increasing aeration increases carbon flux to the acetone/2,3-BDO pathway. Concentrations are calculated as an average of three biological replicates. **(B)** 2,3-BDO concentrations in strain LS203 and LS205 with (pMTL-BD3) and without (pMTL-BD3b) overexpression of the native *bdh*. Overexpression of *bdh* increased 2,3-BDO concentrations across three aeration conditions (3 mL, 5 mL and 10 mL fermentation volumes: bar 1–3, left to right, in each strain). Concentrations are calculated as average of three biological replicates.

It has been shown that both increasing oxygen supply and over-expression of BDH can enhance 2,3-BDO production (Kim et al., 2016). Accordingly, we explored the effect of aeration by varying fermentation volume, using 2 mL, 3 mL and 5 mL. Data obtained indicated a general increase in carbon flux of the acetoin/2,3-BDO pathway with decreasing fermentation volume. More than 60 mM (acetoin + 2,3-BDO) was obtained from 3 mL cultures compared to less than 20 mM across all five *ΔadhE* strains. In terms of 2,3-BDO production, the highest titre achieved was 34 mM, using strain LS205 habouring pMTL-BD3. With more aeration, production switched to acetoin, and in 2 mL cultures up to 52 mM was produced (Figure 5A).

To assess the effect of overexpressing *bdh* we placed it under the control of the native *rplS* promoter and the *G*. *stearothermophilus pheB* RBS (ribosome binding site), which is reported to give strong, constitutive expression levels under varied growth conditions (Reeve et al., 2016). The *bdh* gene was firstly fused with the promoter-RBS and then ligated onto pMTL-BD3 between the XhoI and NheI sites to generate pMTL-BD3b. To circumvent toxicity issues in *E. coli*, the ligation product was transformed directly into the appropriate *P. thermoglucosidasius* NICMB 11955 strain following de-ionizing dialysis treatment and correct transformants were verified by colony PCR and Sanger sequencing (Supplementary Figure S3). Increases in 2,3-BDO production were observed under the three fermentation conditions tested. The highest increase occurred in a 5 mL fermentation volume, with up to 40 and 61 mM being were obtained with LS203 and LS205 carrying pMTL-BD3, respectively. This represented a 1.3-fold (LS203) and 2-fold (LS205) increase (Figure 5B). However, when tested in strain LS105 (which does not carry the *adhE* deletion) under limited aeration, the levels of 2,3-BDO decreased. There was also a 2-fold decrease in ethanol produced and glucose consumed (Data not shown). We concluded that this is because increased *bdh* expression shifts the carbon flux towards the acetoin/2,3-BDO pathway, but without sufficient aeration, NAD^+^ deficiency brings about growth termination. Clearly, 2,3-BDO levels are determined by the intricate balance of oxygen supply and acetoin conversion. To optimize further, a wider range of fermentation volumes were analyzed, and at 6 mL, the highest amount of 2,3-BDO was produced at 63.4 mM (Table 3).

**TABLE 3:** Key metabolite profiles of engineered strains with (LS205) and without (LS206) an *ack* deletion under different fermentation volumes.

Metabolite strains/volume (mL)	% Glucose used		2,3-BDO (mM)		2,3-BDO yield (g/g)		Acetate (mM)		Acetoin (mM)	
	205b	206	205b	206	205b	206	205b	206	205b	206
3	100%	—	29.6 ± 2.0	—	0.13 ± 0.01	—	19.6 ± 2.0	—	46.6 ± 1.7	—
4	97%	—	46.9 ± 7.6	—	0.22 ± 0.03	—	13.8 ± 0.5	—	25.4 ± 0.8	—
5	94%	100%	61.1 ± 1.6	52.0 ± 3.4	0.30 ± 0.01	0.24 ± 0.02	12.2 ± 0.8	13.8 ± 0.8	17.4 ± 5.1	18.6 ± 4.7
5.5	92%	100%	62.0 ± 0.6	54.5 ± 2.9	0.31 ± 0.00	0.25 ± 0.01	12.8 ± 0.8	13.1 ± 1.5	13.1 ± 1.4	12.4 ± 1.5
6	94%	100%	63.4 ± 1.6	60.5 ± 2.9	0.31 ± 0.02	0.27 ± 0.01	11.1 ± 1.0	10.2 ± 1.4	9.4 ± 1.1	6.0 ± 2.1
6.5	94%	100%	59.2 ± 1.6	59.6 ± 0.6	0.29 ± 0.01	0.27 ± 0.00	9.3 ± 0.1	9.3 ± 1.3	5.7 ± 3.1	4.2 ± 2.3
7	91%	98%	55.9 ± 3.3	59.0 ± 1.3	0.28 ± 0.00	0.27 ± 0.00	10.2 ± 0.9	8.5 ± 0.6	2.7 ± 1.7	1.9 ± 0.8
7.5	91%	98%	56.6 ± 2.5	58.9 ± 0.5	0.28 ± 0.00	0.27 ± 0.00	10.3 ± 0.4	8.0 ± 0.4	1.3 ± 0.1	1.4 ± 0.1
8	53%	93%	37.6 ± 6.2	59.3 ± 2.3	0.32 ± 0.03	0.29 ± 0.00	10.7 ± 1.8	8.1 ± 0.2	0.9 ± 0.1	1.5 ± 0.1
8.5	54%	86%	39.0 ± 4.7	55.9 ± 3.8	0.33 ± 0.03	0.29 ± 0.00	8.9 ± 2.7	7.3 ± 0.3	0.8 ± 0.1	1.3 ± 0.1
9	73%	77%	47.4 ± 5.2	50.6 ± 1.0	0.30 ± 0.01	0.30 ± 0.01	9.7 ± 0.4	6.8 ± 0.3	1.1 ± 0	1.2 ± 0.1
10	78%	72%	50.4 ± 1.1	49.2 ± 1.2	0.29 ± 0.00	0.31 ± 0.00	8.5 ± 0.4	6.8 ± 0.3	0.9 ± 0	1.2 ± 0.1
206b										
6	98%		72.6 ± 1.8		0.33 ± 0.01		13.6 ± 0.9		25.9 ± 3.4	

Cells either carrying pMTL-BD3 (not indicated) or pMTL-BD3b (indicated as b) are cultured for 48 h in M-ASYE, with 2% glucose and yeast in 50 mL falcon, shaking at 250 rpm, 50°C before product measurement. Error bars represent standard deviation of three biological replicates.

### 3.6 Identification of an acetoin utilization gene

In acetoin-excreting bacteria, acetoin catabolism can occur via the lipoic acid-mediated conversion of acetoin to acetaldehyde, allowing the re-utilization of the 4 carbon compound for energy (Xiao and Xu, 2007). Acetoin utilization depletes carbon flux from the acetoin/2,3-BDO pathway. It follows that measures that disrupt acetoin utilization, either through deletion of catalytic component or transcriptional regulators, can lead to increased acetoin levels (Zhang et al., 2016; Li et al., 2017a).

To test whether *P. thermoglucosidasius* NICMB 11955 can catabolise acetoin, we grew the WT strain in defined medium containing 1% (w/v) acetoin or 2,3-BDO as the sole carbon source in baffled-flasks at 60°C. NICMB 11955 utilized both acetoin and 2,3-BDO rapidly, reaching a maximum OD_600_ of >8.0 in 12 h. A protein BLAST (BLASTp) search of the *P. thermoglucosidasius* genome identified three potential candidates involved in catabolism. The first candidate AcuABC (locus BCV53_18410-18420) is annotated as acetoin utilization gene ABC and comprises a polycistronic operon with AcuA separated by 34 bp from AcuB and AcuB overlapped by 4 bp with AcuC. The further two candidates were annotated as (acetyl-transferring) pyruvate dehydrogenase complex (PDHC) E1 component subunits (BCV53_10285 and 10935).

The AcuABC proteins share 80%, 55% and 70% sequence identity with the *B*. *subtilis* acetoin utilization proteins, AcuA (ACDH), AcuB, and AcuC (histone deacetylase), one of two separate operons (AcuABC and AcoABCL) responsible for acetoin consumption in this *bacillus* (Grundy et al., 1993; Ali et al., 2001). Interestingly, 88 bp 3’ of the *P. thermoglucosidasius* NICMB 11955 *acuC* gene is the a gene coding for the catabolite control protein A (CCPA), which in *B. subtilis* was reported to negatively regulate acetoin utilization by repression of AcoR, the transcriptional activator of AcuABC (Ali et al., 2001; Peng et al., 2020).

The two identified PDHCs belong to the 2-oxoacid hydrogenase complex (OADHCs.) family, a super-family containing many different dehydrogenase complexes, including the acetoin dehydrogenase system (ADHC) (Perham, 2003). The NCBI annotation pipeline is based upon sequence identity and domain predictions (Tatusova et al., 2016), therefore annotation can be misleading. On closer inspection, both PDCs showed sequence identity (75% and 46%) to *B. subtilis* Acetoin:2,6-dichlorophenolindophenol (DCPLP) oxidoreductase subunit alpha. A study by Oppermann et al. (1991), suggests that the alpha subunit is likely the catalytic subunit responsible for the cleavage of acetoin into acetate and acetaldehyde. Hence, we decided to test this hypothesis by deleting the encoding genes, using CRISPR/Cas, and assessing the effect on acetoin utilization. For the purpose of this study we renamed the two PDHC (BCV53_10285 and 10935) as AcoB1 and AcoB2.

**FIGURE 6:**
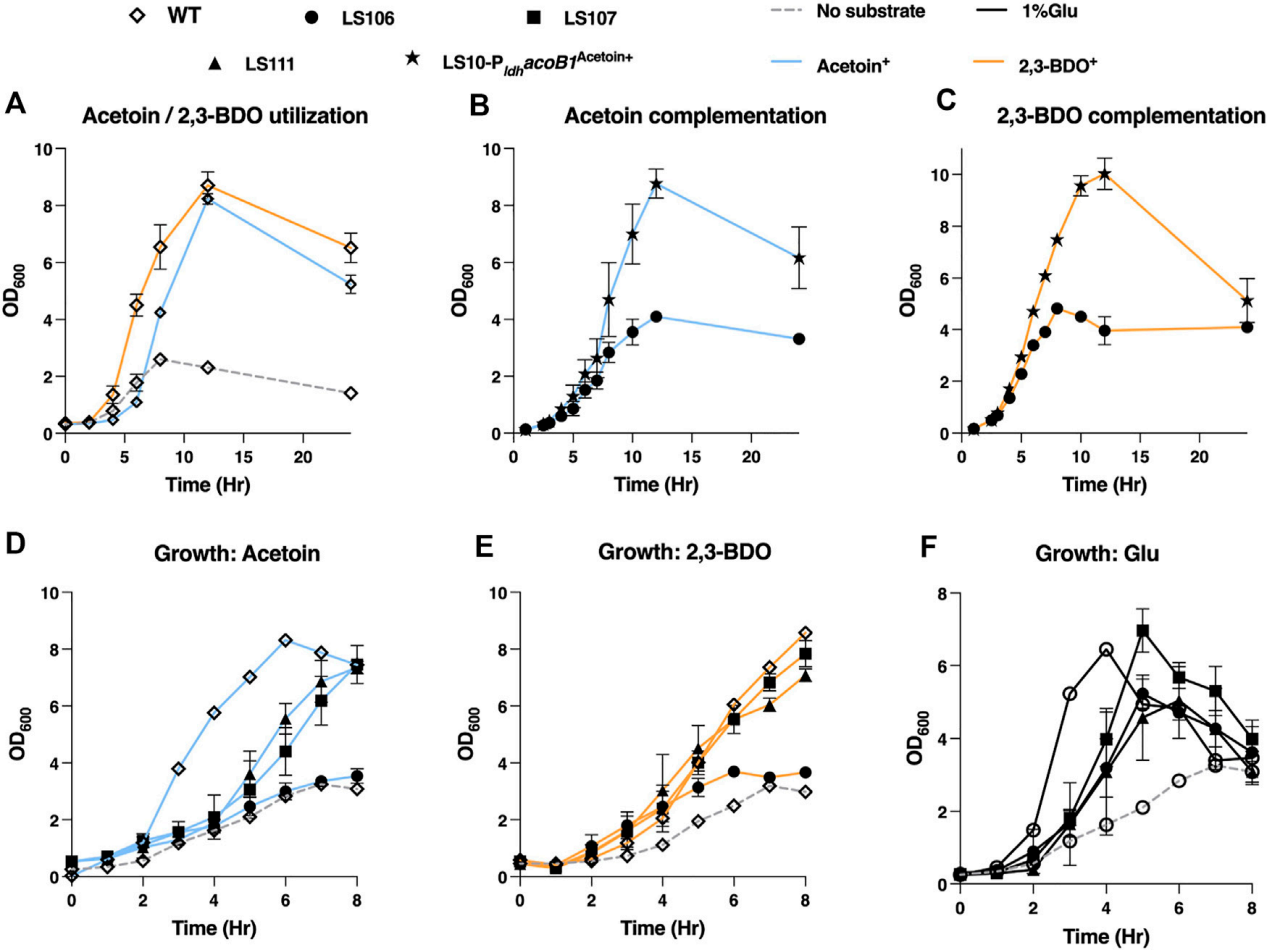
Testing the ability of *P. thermoglusidasius* NCIMB 119 to consume acetoin and/or 2,3-BDO. **(A)** Growth curve of *P. thermoglusidasius* NCIMB 11955 on acetoin (AC) and 2,3-BDO (BDO) as the sole source of carbon. **(B)** Growth on acetoin of NCIMB 11955 deletion mutants *ΔacoB1* (LS106), *ΔacoB2* (LS107) and *ΔacuA-C* (LS111). **(C)** Growth on 2,3-BDO of LS106, LS107 and LS111. **(D)** Growth of LS106, LS107 and LS111 on glucose. **(E)** Complementation of LS106 growth on acetoin through plasmid-based overexpression the *acoB1* gene. **(F)** Complementation of LS106 growth on 2,3-BDO through plasmid-based overexpression the *acoB1* gene. In all cases, time zero = inoculation of overnight liquid culture to OD_600_ of 0.25–0.5. Cells were propagated at 60°C in CBM with a 1% (w/v) carbon source and shaking (250 rpm) in 250 mL baffled flasks. CBM without a carbon source was used as negative control. Error bars represent the standard deviation of three biological replicates.

To counter acetoin utilization the mutant strains generated were LS106 (Δ*acoB1*), LS107 (Δ*acoB2*), LS108 (Δ*acuA*), LS109 (Δ*acuB*), LS110 (Δ*acuC*) and LS111 (Δ*acuA-C*). Deletion of *acoB1* had a dramatic effect, with no growth evident on media containing either acetoin or 2,3-BDO as the sole carbon source. In contrast, all other mutants had a similar growth profile to the WT. Using glucose, slower growth was observed with all mutants, and the maximum OD_600_ of LS106 (Δ*acoB1*) and LS111 (Δ*acuC*) were slightly reduced compared to WT. For further validation, we expressed the AcoB1 gene in the Δ*acoB1* strain under the control of the P_*ldh*_ promoter using the expression plasmid pMTL61110_pldh. Complementation of the WT growth profile were observed for both acetoin and 2,3-BDO (Figure 6).

### 3.7 Enhanced acetoin production using *ΔacoB1* strains

Following the identification of the acetoin utilization gene *acoB1*, it was knocked out in the five Δ*adhE* strains generating five replicate, double knock-out mutants designated LS300, LS301, LS303, LS304 and LS305 (Table 2). These were transformed with plasmid pMTL-BD3, grown in 2 mL fermentation volumes and the acetoin and 2,3-BDO levels measured. A significant increase was observed, with up to 97 mM (acetoin + 2,3-BDO) produced after 48 h, representing 87% of all carbon flux. While 2,3-BDO levels remained at around 10 mM in all five strains, acetoin production was greatly enhanced with the highest titre of 7.7 g/L (87 mM) in strain LS305, equating to a yield of 0.38 g/g glucose (78% TMY) (Table 4).

**TABLE 4:** Comparison of key metabolite profiles of LS200 (*ΔadhE*) strains carrying pMTL-BD3 with and without deletion of *acoB1* under aerobic conditions.

Strains	Acetoin (mM)			Acetoin yield (g/g)		% Increase	2,3-BDO (mM)		Acetate (mM)	
	*acoB1* ^*+*^	*ΔacoB1*	*acoB1* ^*+*^		*ΔacoB1*	*-*	*acoB1* ^*+*^	*ΔacoB1*	*acoB1* ^*+*^	*acoB1*
LS200	46.5 ± 0.1	54.9 ± 3.8	0.20		0.24	18.1	10.4 ± 1.0	15.1 ± 2.5	21.0 ± 2.1	13.0 ± 1.9
LS201	51.4 ± 3.1	77.2 ± 10.1	0.23		0.34	50.0	8.0 ± 1.9	15.0 ± 2.6	19.9 ± 1.1	7.4 ± 0.7
LS203	45.0 ± 1.2	73.2 ± 5.4	0.20		0.32	62.7	10.8 ± 0.7	13.4 ± 1.0	19.9 ± 2.0	7.1 ± 0.2
LS204	44.6 ± 3.8	69.3 ± 8.6	0.20		0.31	55.5	10.1 ± 0.1	8.4 ± 4.9	23.4 ± 5.1	8.3 ± 1.3
LS205	43.7 ± 2.7	87.6 ± 1.5	0.19		0.39	100.4	11.9 ± 0.8	10.5 ± 3.3	23.7 ± 1.6	10.4 ± 4.8

Strains were cultured for 48 h in 2 mL of M-ASYE, media containing 2% (w/v) of glucose and yeast extract in 50 mL falcon with shaking (250 rpm) at 50°C. error bars represent standard deviation of three biological replicates.

With nearly 90% of the carbon entering the acetoin/2,3-BDO pathway, we hypothesized that abolishing the production of 2,3-BDO by deletion of *bdh* would further enhance acetoin production. The *bdh* gene was therefore knocked in the five Δ*acoB1/*Δ*adhE* double mutant strains generating strains LS400, LS401, LS403, LS404 and LS405 (Table 2). Production of 2,3-BDO in these strains was entirely abolished, however, acetoin titres also decreased. To investigate whether oxygen supply was the limiting factor, baffled shake-flasks were used in place of falcon-tube concentration (Supplementary Figure S2). Acetoin titres, however, remained essentially the same.

### 3.8 Disruption of acetate kinase

Thus far, for both acetoin and 2,3-BDO production, acetate was the only by-product measured that was still produced in significant amounts. In other studies, disruption of acetate production has been shown to enhanced 2,3-BDO yields (Jantama et al., 2015). Accordingly, the genes encoding phosphate acetyltransferase (*pta*) (BCV53_03505) and acetate kinase (*ack*) (BCV53_18310) were targeted for deletion in strains LS205 and LS305. Only *ack* mutants, LS206 and LS306, were obtained. Both strains were transformed with pMTL-BD3 to allow investigation of the effect of the *ack* mutation on acetoin/2,3-BDO production.

LS306 (pMTL-BD3) failed to grow under the conditions tested. This may be partly due the level of acetyl-CoA in this strain being insufficient. Acetyl-CoA can be reassimilated through the AcDH-mediated conversion of acetoin to acetaldehyde using cofactor CoA and NAD^+^. Acetaldehyde is further oxidised to acetyl-CoA through the activity of acetaldehyde dehydrogenase (AldDH) (Wolfe, 2005; Extance et al., 2016). Analysis of the *P. thermoglucosidasius* NICMB 11955 genome identified four putative genes encoding an AldDH, namely, BCV53_01645, BVC53_06570, BVC53_ 12995 and BVC53_14470. Alternatively, acetyl-CoA re-assimilation can occur either via the combined action of PtA and AckA or through acetyl-CoA synthetase (ACS) (Pinhal et al., 2019; Meng et al., 2021). Since most routes from pyruvate to acetyl-CoA are disrupted in our strain, and with carbon fluxes diverted towards the acetoin/2,3-BDO pathway, the re-assimilation pathways might be of vital importance to provide cells with sufficient acetyl-CoA for the TCA cycle and biosynthesis.

**FIGURE 7:**
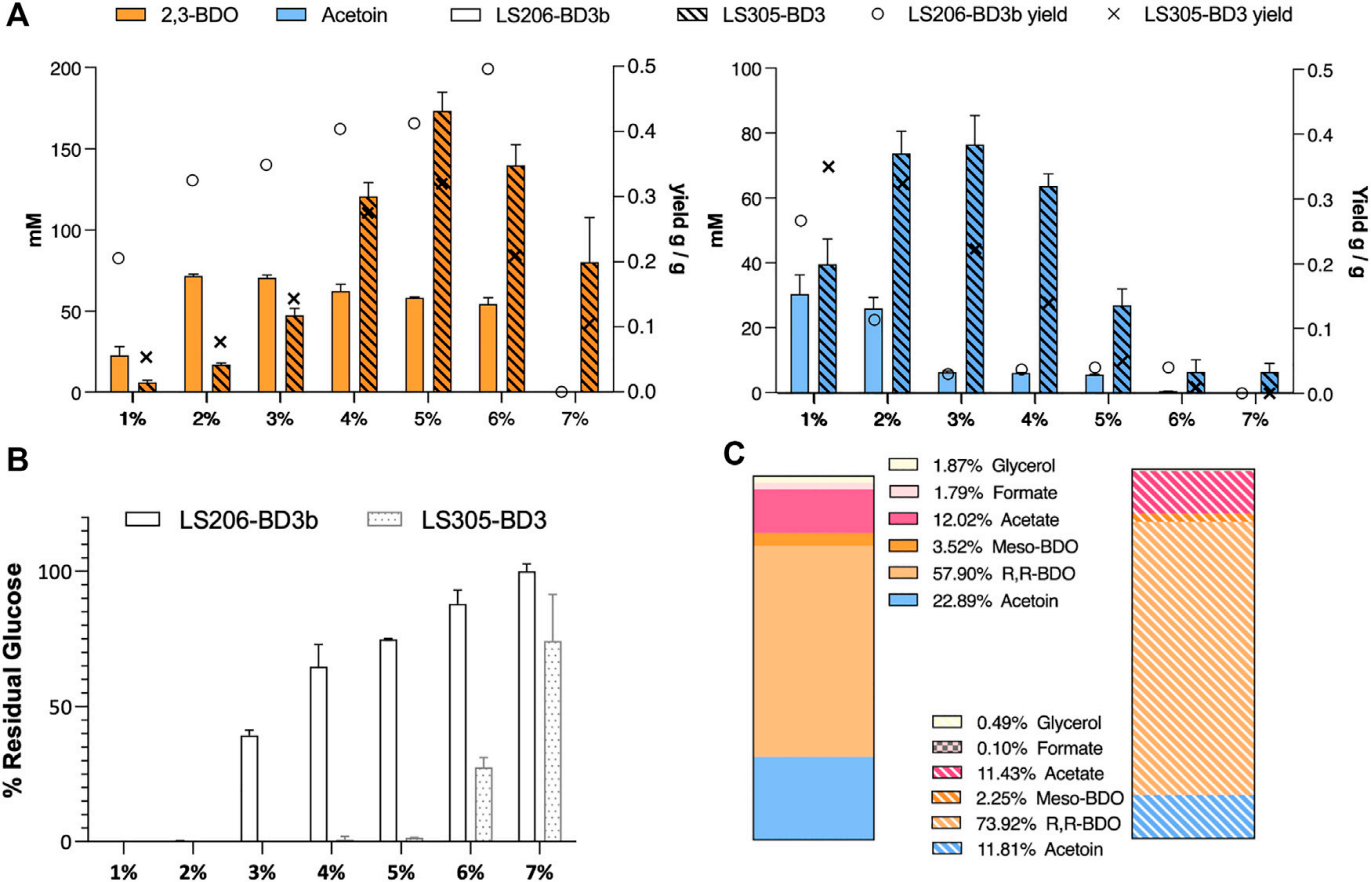
The effect of glucose concentration on acetoin and 2,3-BDO production. **(A)** Acetoin and 2,3-BDO levels under different glucose concentrations. Yield (right Y-axis) is gram of product produced per gram of glucose consumed. 2,3-BDO yields increase while acetoin yields decrease with higher glucose concentrations. More glucose was consumed under more aerated condition (2 mL fermentation volume, strain LS305 habouring pMTL-BD3), and less under oxygen limitation (6 mL fermentation volume, LS206 habouring pMTL-BD3b) **(B)** At 7% glucose and above, cells became unviable. Error bars represent the standard deviation of three biological replicates. **(C)** Percentage product profiles of strain LS206 [pMTL-BD3b] grown at 50°C for 48 h, with shaking (250 rpm), in 6 mL M-ASYE with 2% (w/v) glucose (plain) and LS305 [pMTL-BD3] in 2 mL M-ASYE with 5% (w/v) glucose (stripped). The majority of carbon flux was in the acetoin/2,3-BDO pathway (84% and 88%) with 2,3-BDO being the predominant product (61.4% and 76%). Values are the means of three biological replicates. BDO = 2,3-butanediol and AC = Acetoin.E.

In LS206 (pMTL-BD3), 2,3-BDO the titre was comparable to that of LS205 habouring pMTL-BD3b (LS205b) when using 5–7.5 mL fermentation volumes, and much higher under more oxygen-limited conditions (Table 3). However, 2,3-BDO yields were slightly lower in LS206 (pMTL-BD3) compared to LS205b and more glucose was consumed for similar amounts of 2,3-BDO produced. Despite disruption of *ack*, acetate was still present, though at a slightly lower level compared to LS205b, probably due to the presence of alternative routes to its formation, involving, for instance, acetyl-CoA ligase (Pineda et al., 2016) and acetyl-CoA hydrolase (Lee et al., 1996), both of which are present in the *P. thermoglucosidasius* NICMB 11955 genome (BCV53_18295; BCV53_18405 and BCV53_05785). Next, 2,3-BDO level was measured in LS206b. Again, the decoupling of *bdh* expression positively affected 2,3-BDO production, with up to 73 mm obtained, representing 0.33 g/g yield, the highest yield obtained in this study and 66% of the TMY. In addition, up to 25.9 mM acetoin was obtained, indicating that up to 89% of total carbon flux went into the acetoin/2,3-BDO pathway (Figure 7C).

### 3.9 Effect of glucose concentration on acetoin and 2,3-BDO production

Finally, we sought to investigate the effect of different concentrations of glucose on our engineered strains. Not only it is important that the strains can tolerate a range of glucose concentrations for industrial flexibility, different glucose concentrations tend to introduce significant changes in the cell physiology such as increased osmotic stress (Gonzalez et al., 2016), inhibition and activation of certain metabolic pathways (Guidi et al., 2010). In general, glucose concentration positively affected 2,3-BDO titres but hampered acetoin production. In LS206 (pMTL-BD3b), for instance, using 6 mL fermentation volumes, a near linear relationship in 2,3-BDO yield was observed with increasing glucose concentration, with up to 0.5 g/g in 6% glucose, representing near 100% TMY. However, 2,3-BDO titres plateaued at 2% glucose, with less glucose being utilized above this concentration. It seems that excessive glucose negatively affects catabolism. In LS305 (pMTL-BD3), the use of small (2 mL) culture volume alleviated the constraints on glucose utilization, which was completely utilized at concentrations up to 5% glucose. Increases in 2,3-BDO yield mirrored the trend seen with LS206 (pMTL-BD3b), with titres peaking at 0.32 ± 0.2 g/g when the glucose concentration was 5%, representing a titre of 15.6 ± 1.4 g/L, the highest reported in *Geobacillus* (Figure 7).

## 4 Discussion

Acetoin and 2,3-BDO are biologically related chemicals of significant commercial interest. Their bioproduction using microorganisms may provide a more carbon neutral alternative to the traditional petrochemical routes. Here, through rational metabolic engineering, the thermophilic chassis *P. thermoglucosidasius* NCIMB 11955 has been established as a platform organism for bioproduction of both acetoin and 2,3-BDO.

Although previous work has demonstrated the production of 2,3-BDO in *P. thermoglucosidasius* DSM 2542 (Zhou et al., 2020), the authors reported inherent problems with redox imbalance and failed to solve the issue of by-product (ethanol) formation. It has been proposed that the physiological role of the 2,3-BDO pathway is to supply NADH for elimination of the toxic acetate produced during overflow metabolism, channelling reducing power to ethanol production (Meng et al., 2021). This suggests the formation of 2,3-BDO and ethanol occurs at a 1:1 ratio, which is what we observed in all the *adhE* mutants carrying the 2,3-BDO pathway. Disruption of the ethanol pathway resulted in increased acetate titres and decreased 2,3-BDO production. It is important to minimize by-product formation, as not only is it wasteful of substrate, it may also complicate downstream extraction adding to processing costs. We confronted the redox issue through adjusted aeration and decoupling the expression of the native *bdh*, which we identified and characterized. Along with disruptions of genes targeting metabolites of the mixed fermentation pathway (including lactate, glycerol, formate and acetate) 2,3-BDO is produced as the major product at 6.6 g/L, representing a yield of 0.33 g/g and 66% of TMY. Although this failed to exceed the 7.2 g/L titre in *P. thermoglucosidasius* DSM 2542 reported previously (Zhou et al., 2020), ethanol production is completed eliminated, and only acetate and acetoin levels exceeded 10 mM. Amongst all the metabolites measured, 61% was 2,3-BDO, of which 94% was the R,R-enantiomer. Along with 23% acetoin, carbon flux to the acetoin/2,3-BDO route altogether represents 84% of total metabolites, with 89% carbon from glucose converted, giving scope for further improvement.

Product utilization was also investigated. Many bacterial species, including *B. subtilis, Micrococcus urea, Alcaligenes eutrophus, Enterococcus faecalis, Pelobacter carbinolicus, Klebsiella pneumoniae* and *Clostridium magnum,* are able to degrade acetoin (Ali et al., 2001). Acetoin catabolism in *P. thermoglucosidasius*, and thermophilic microorganisms in general, has not been described. Within any bioprocesses, it is important to ensure that the generated compounds are not re-metabolised by the producing organisms (Arenas-López et al., 2019). Unfortunately, NCIMB 11955 was found to utilize both acetoin and 2,3-BDO as sole carbon source for growth, a property that required elimination.

Two independent pathways of acetoin catabolism have been proposed in B. *subtilis*, one involving the AcuABC operon (Grundy et al., 1993) and the other involving the Aco operon encoding the multicomponent acetoin dehydrogenase complex (Huang et al., 1999). Putative genes for both pathways are present in the NCIMB 11955 strain, but acetoin appears to be catabolized solely via the acetoin dehydrogenase (AcDH) route, as deletion of the AcuABC operon in failed to prevent acetoin utilization, while a single deletion of the AcoB1 gene abolished growth on both acetoin and 2,3-BDO.

AcDH belongs to the family of 2-oxo acid dehydrogenase complexes which uses the reducing power of NAD^+^ to convert 2-oxo acids to the corresponding acyl-CoA derivatives and produce NADH and CO_2_ (De Kok et al., 1998). During 2,3-BDO fermentation, when oxygen is limiting and NAD^+^ are not recycled due to only one produced via the acetoin/2,3-BDO route, AcDH is likely inactive. However, with more aeration, NAD^+^ availability would be sufficient to drive acetoin catabolism, which can affect product titre. Therefore, we hypothesized that deletion of the AcoB1 gene would have profound implications in more aerobic conditions and less or no effect under oxygen limitation, hence more relevant for acetoin production. Data using the *acoB1* mutant supports this: at both 5 mL and 10 mL fermentation volumes, 2,3-BDO produced was comparable in strains LS205 (without *acoB1* deletion) and LS305 (with *acoB1* deletion). However, when using 2 mL, acetoin production increased from 43.7 ± 2.7 mM to 86.0 ± 0.1 mM, respectively, for the same strains, representing a near 100% increase in titre.

Further strategies to enhance acetoin production, including deletion of *bdh,* and varied glucose concentration were unsuccessful. Deletion of the native *bdh* gene resulted in lower acetoin production levels, while its production decreased proportionally to glucose concentration, suggesting that unknown mechanisms such as osmotic stress can regulate 2,3-BDO and acetoin biosynthesis. Remarkably, under aerobic conditions, the predominant metabolite in LS305 shifted from acetoin to 2,3-BDO with increasing glucose concentration, amounting to 15.6 g/L at 5% glucose, representing 64% TMY. The yield obtained here is comparable to what was achieved with LS206 (pMTL-BD3b), (0.32 g/g vs. 0.33 g/g), with the benefit of being able to metabolize more glucose. Moreover, carbon fluxes to the acetoin/2,3-BDO pathway is also comparable, if not better, compared to LS206 (pMTL-BD3b), (88% vs. 84% total metabolites), with 76% being 2,3-BDO, of which 97% is the R-R enantiomer (Figure 7C). These data underline the benefit of incorporating a *acoB1* deletion mutation in strains to support high 2,3-BDO titres under more aerated conditions.

## 5 Conclusion

In conclusion, we have created platform strains for the production of acetoin and 2,3-BDO at thermophilic temperatures. By addressing issues of redox imbalance and product utilization, substantial 2,3-BDO was produced with minimal by-product formation. In parallel, for the first time in the industrially relevant strain *P. thermoglucosidasius* NCIMB 11955 we demonstrated high yields of acetoin, on par with the highest reported in the literature. This work also serves as a foundation for the optimization of fermentation at a larger scale, with much potential for further improvement.

## Data Availability

The original contributions presented in the study are included in the article/Supplementary Material, further inquiries can be directed to the corresponding author.
