# Supplementary material for: Metabolic engineering for the production of acetoin and 2,3-butanediol at elevated temperature in Parageobacillus thermoglucosidasius NCIMB 11955
Source: Front Bioeng Biotechnol. 2023 May 2;11:1191079. doi: 10.3389/fbioe.2023.1191079 (PMC10185769; doi:10.3389/fbioe.2023.1191079)
Supplement: Supplementary file 1 [file DataSheet1.docx]

***Supplementary Material***

**Metabolic Engineering for the production of acetoin and 2,3-butanediol at elevated temperature in *Parageobacillus thermoglucosidasius* NCIMB 11955**

**Lili Sheng, Abubakar Madika, Matthew S.H. Lau, Ying Zhang, Nigel P. Minton^*^**

* **Correspondence:** Nigel P. Minton: nigel.minton@nottingham.ac.uk

**SUPPLEMENTARY FIGURES**

**
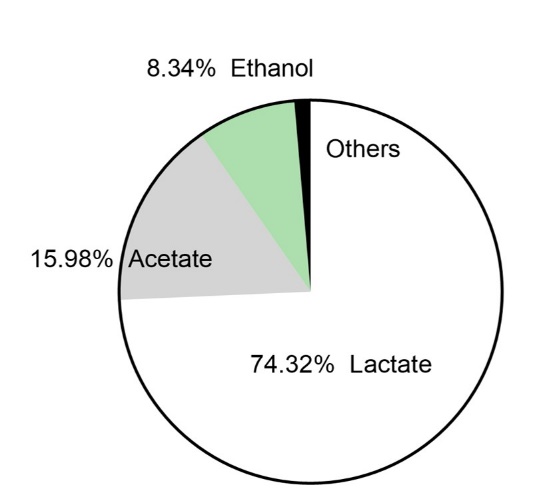
**

**Figure S1.** Typical metabolites produced by *P.* *thermoglucosidasius* NICMB 11955. Overnight cultures were grown in 10ml 2SPYNG media in 50 ml Falcon tube shaking at 60 °C for 48 hours before measurement. The major metabolite produced is lactate (white) with some acetate (grey) and ethanol (green). Acetoin and 2,3-butanediol are not detectable.

**
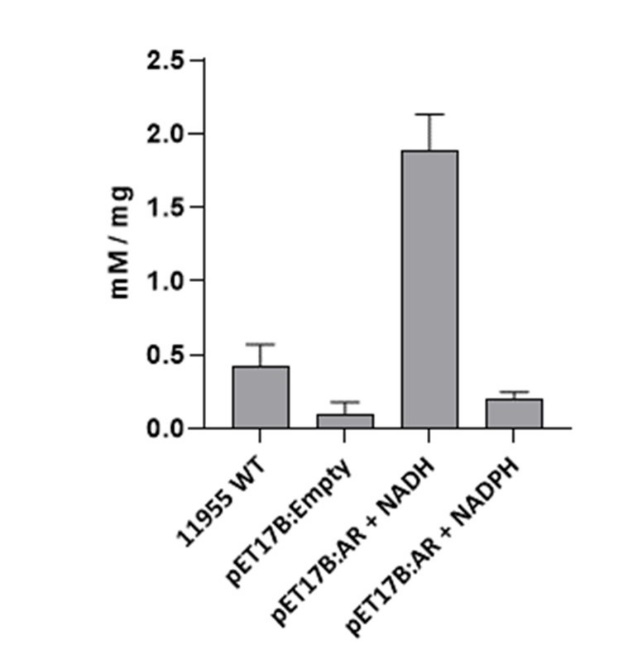
**

**Figure S2.** Confirmation that the BDH protein of *P.* *thermoglucosidasius* NICMB 11955 has acetoin reductase (AR) activity. Spectrophotometric assay of lysates prepared from the *P.* *thermoglucosidasius* NICMB 11955 WT (column 1) and *E. coli* strain BL21 (DE3) carrying either insert-less pET17b (column 2) or pET17b with a cloned copy of the *bdh* assayed with NADH (column 3) or NADPH (column 4). The axis represents amount of NADH turnover per mg of protein in the crude cell lysate. Error bars represent standard deviation, n=3. Lane 1, WT, plasmid-free.

**
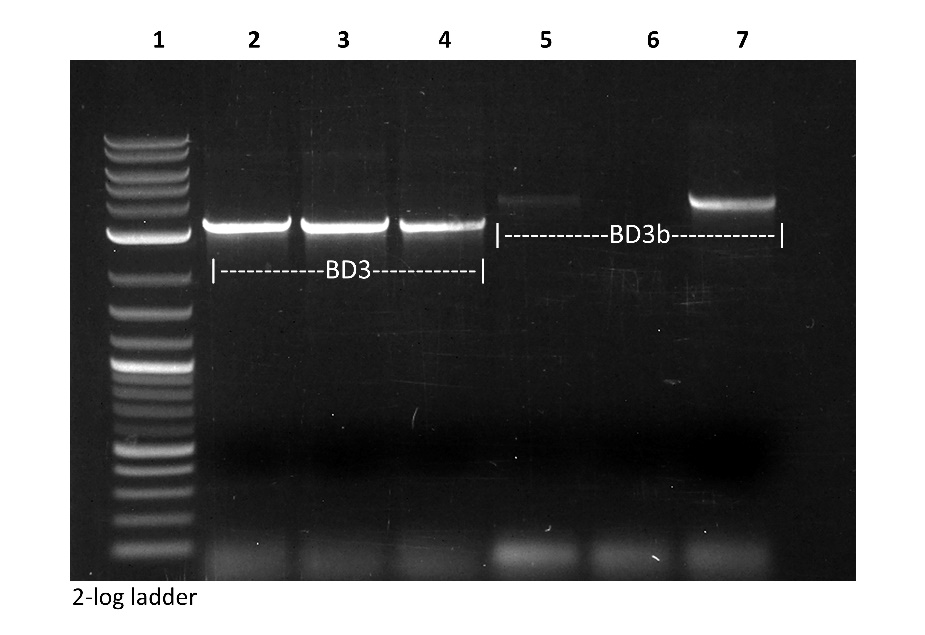
**

**Figure S3.** Direct cloning of the *bdh* gene into P. *thermoglucosidasius*. Gel electrophoresis image of a colony PCR, utilizing pLDH_Seq and RepB_R1 primers, to assess whether the native *bdh* gene has been successfully ligated in the XhoI / NheI site of pMTL-BD3 in P. *thermoglucosidasius*. Successful ligation (BD3b) would yield a band of ∼4.5 kb (lane 5-7) whereas transformants with pMTL-BD3 is used as a control (∼3.3kb, lane 2-4). A 2-log DNA ladder was used to determine the size of each band (Lane 1).


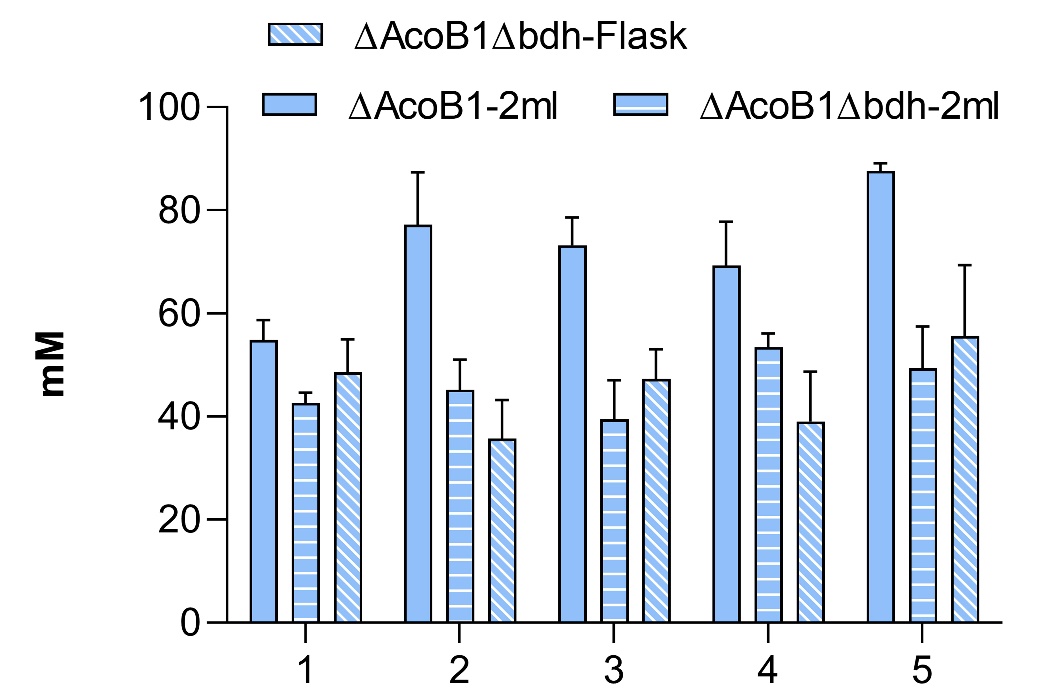


**Figure S4.** Comparison of acetoin levels after 48 hours shaking at 250 rpm, 50⁰C, under aerobic conditions for *ΔacoB1* strains with and without a *bdh* gene deletion. Group 1 – 5 represents deletions referred in strains LS200, LS201, LS203, LS204 and LS205. Lack of BDH failed to enhance acetoin production both when grown in 2 ml of media in a 50 ml falcon tube or baffled flask.

**SUPPLEMENTARY TABLES**

**Table S1:** Microbial 2,3-butanediol producers

| **Organism** | **Genotype and modifications** | **Fermentation Condition** | **Yield (g / g glucose)** | **Source** |
| --- | --- | --- | --- | --- |
| *Saccharomyces* ***cerevisiae*** | Strain JUK36α: {*delta1-FBA1p-alsS-PGIt-PDC1p-alsDADH2t-THD3p-BDH1-CYC1t- EGFP-delta2*} based on delta integration enhanced by CRISPR; ADH1, PDC1, PDC5, MTH1 disruption based on CRISPR | Fed-batch, 30⁰C, pH 5.5 | 0.17 | (Huang and Geng, 2020) |
| *Saccharomyces* ***cerevisiae*** | Strain D452‑2: *pdc1Δpdc5Δpdc,6p423_alsSalsD*, p425_BDH1 p406GPD2_CtPDC1, pAUR_Llnox | Fed-batch, 30⁰C, pH 5.5 | 0.404 | (Kim *et al.,* 2016) |
| *Escherichia coli* | Strain DSM1116: *∆ldhA∆adhE∆pta∆frdA∆pykA, budAbudBbudC* | Fed-batch, 37⁰C, pH 7.0 | 0.38 | (Erian *et al.,* 2018) |
| *Corynebacterium crenatum* | *∆ldh; alsS-alsD-bdhA* | Batch, 30⁰C, pH 7.0 | 0.40 | (Zhang *et al.,* 2019) |
| *Corynebacterium glutamicum* | ATCC13032 *ΔaceEΔpqoΔldhAΔmdh*, (pEKEx2-*als, aldB*,PtufbutA) | Fed-Batch, 30⁰C | 0.33 | (Radoš *et al.,* 2015) |
| *Pichia pastoris* | Strain X33: integration of AlsS and AlsD | Flask, 37⁰C, pH 5.0 | 0.30 | (Yang and Zhang, 2018b) |
| *Escherichia coli* | Strain MG1665: pUC6S-AlperP_LTet01_-*budB-budA-*AlperP_LTet01_-*ydjL* | Fed-batch, 37⁰C, pH 7.0 | 0.38 | (Tong *et al.,* 2016) |
| *Klebsiella oxytoca* | Strain M1; pUC18CM-*bud*C | Fed-batch, 30⁰C, pH 6.0 | 0.42 | (Cho *et al.,* 2015) |
| *Klebsiella oxytoca* | Strain ME-UD3: *ald*A::*Tcr* | Fed-batch, 37⁰C, pH 6.5 | 0.48 | (Ji *et al.,* 2010) |
| *Klebsiella oxytoca* | Strain M5a1: *ΔadhEΔackA-ptaΔldhA:cat-sacB* | Fed-batch, 37⁰C, pH 6  Shake Flask | 0.49  0.46 | (Jantama *et al.,* 2015) |
| *Bacillus subtilis* | Strain 168: *alsSD-bdhA*, | Batch, 37⁰C, pH uncontrolled | 0.34 | (Biswas *et al.,* 2012) |
| *Bacillus amyloliquefaciens* | Strain B10-127 | Batch, 37⁰C, pH 6.5 | 0.42 | (Yang *et al.,* 2015) |
| *Bacillus licheniformis* | - | Shake Flask, 37⁰C, pH 6.0 | 0.47 | (Nilegaonkar *et al.,* 1992) |
| *Paenibacillus polymyxa* | Strain DSM365: *Δldh1 pHEiP_Ppbdh* | Continues, 35⁰C, pH 6.0 | 0.43 | (Schilling *et al.,* 2020) |

**Table S2:** Microbial acetoin producers

| **Organism** | **Genotype and modifications** | **Fermentation Condition** | **Yield (g / g glucose)** | **Source** |
| --- | --- | --- | --- | --- |
| *Saccharomyces* ***cerevisiae*** | Strain JHY605: *Δadh1-5Δgpd1Δgpd2Δbdh1; alsS-alsD-noxE* | Batch  Shake Flask | 0.44  0.39 | (Bae *et al.,* 2016) |
| *Escherichia coli* | Strain YYC202: *ΔldhAΔilvC pET-ilvB-NEc-aldCLl* | Shake Flask 37⁰C, pH 7.0 | 0.22 | (Nielsen *et al.,* 2010) |
| *Corynebacterium crenatum* | *∆ldh∆butA; alsS-alsD* | Batch, 30⁰C, pH 7.0 | 0.33 | (Zhang *et al.,* 2019) |
| *Enterobacter cloacae* | Strain SDM: *ΔbudCΔfrdAΔldhA; P_5__NOX* | Fed-batch, 37⁰C, pH 7.0 | 0.37 | (Su *et al.,* 2021) |
| *Paenibacillus polymyxa* | Strain CS107 | Fed-batch 37⁰C, pH 7.0 | 0.37 | (Zhang *et al.,* 2012) |
| *Bacillus amyloliquefaciens* | Strain FMME044 – adaptive evolution for acetoin tolerance | Batch, 37⁰C, pH 6.0 | 0.44 | (Zhang *et al.,* 2014) |
| *Bacillus subtilis* | Strain JNA 3-10: *ΔbdhA, yodC* | Batch, 37⁰C, pH 6.8 | 0.38 | (Bae *et al.,* 2016) |

**Table S3:** Plasmids Used

| **Name** | **Relevant Description** | **Source** |
| --- | --- | --- |
| pET17b | *E. coli* expression vector, pBR322, Amp^R^, P_T7_ | Millipore |
| pMTL61110 | GT shuttle vector based on pMTL-series, （RepB, ColE1, Kan^R^） | This study |
| pET17b-bdh | *E. coli* expression vector of native GT *bdh* | This study |
| pMTL61110_pldh | GT expression vector, T_t1t2_- *G. stereothermophilus* P*_ldh_* (P*_ldh_*_(_*_gs_*_)_) | This study |
| pMTL-LS-ALS1 | pMTL61110, P*_ldh_*_(_*_gs_*_)_-*B. subtilis* ALS (*als1*) | This study |
| pMTL-LS-ALS2 | pMTL61110, P*_ldh_*_(_*_gs_*_)_-*B. coagluan* ALS (*als2*) | This study |
| pMTL-LS-ALD1 | pMTL61110, P*_ldh_*_(_*_gs_*_)_-*B. acetylicum* ALD (*ald1*) | This study |
| pMTL-LS-ALD2 | pMTL61110, P*_ldh_*_(_*_gs_*_)_-*B. coagluan* ALD (*ald2*) | This study |
| pMTL-LS-ALD3 | pMTL61110, P*_ldh_*_(_*_gs_*_)_-*B. cereus* ALD (*ald3*) | This study |
| pMTL-LS-ALD4 | pMTL61110, P*_ldh_*_(_*_gs_*_)_-*P. thermopronionicum* ALD (*ald4*) | This study |
| pMTL-LS-ALD5 | pMTL61110, P*_ldh_*_(_*_gs_*_)_-*C. thermophilum* ALD (*ald5*) | This study |
| pMTL-LS-ALD6 | pMTL61110, P*_ldh_*_(_*_gs_*_)_-*T. terretis* ALD (*ald6*) | This study |
| pMTL-LS-BD1 | pMTL61110, P*_ldh_*_(_*_gs_*_)_-als1- P*_gapd_*_(_*_gt_*_)_-ald1 | This study |
| pMTL-LS-BD1t | pMTL61110, P*_ldh_*_(_*_gs_*_)_-als1- P*_gapd_*_(_*_gt_*_)_-ald1t (ald1 5′ truncated 141bp) | This study |
| pMTL-LS-BD2 | pMTL_61110, P*_ldh_*_(_*_gs_*_)_-als2- P*_gapd_*_(_*_gt_*_)_-ald1 | This study |
| pMTL-LS-BD3 | pMTL_61110, P*_ldh_*_(_*_gs_*_)_-als1- P*_gapd_*_(_*_gt_*_)_-ald3 | This study |
| pMTL-LS-BD5 | pMTL_61110, P*_ldh_*_(_*_gs_*_)_-als1- P*_gapd_*_(_*_gt_*_)_-ald5 | This study |
| pMTL675555 | stCas9-3 based CRISPR gene knockout vector | Lau *et al.,* 2021 |
| pCas9-g3pdh1 | Deletion of G3PDH1 based on pMTL675555 | This study |
| pCas9-g3pdh2 | Deletion of G3PDH2 based on pMTL675555 | This study |
| pCas9-gdh1 | Deletion of GDH1 based on pMTL675555 | This study |
| pCas9-gdh2 | Deletion of GDH2 based on pMTL675555 | This study |
| pCas9-ldh2 | Deletion of LDH2 based on pMTL675555 | This study |
| pCas9-adhe | Deletion of *adhE* based on pMTL675555 | This study |
| pCas9-acoB1 | Deletion of *acoB1* based on pMTL675555 | This study |
| pCas9-AcoB2 | Deletion of *acoB2* based on pMTL675555 | This study |
| pCas9-AcuA | Deletion of *acuA* based on pMTL675555 | This study |
| pCas9-AcuB | Deletion of *acuB* based on pMTL675555 | This study |
| pCas9-AcuC | Deletion of *acuC* based on pMTL675555 | This study |
| pCas9-AcuAC | Deletion of *acuAC* based on pMTL675555 | This study |
| pCas9-bdh | Deletion of BDH based on pMTL675555 | This study |

**Table S4:** Primers Used

| **Primer Name** | **Sequence** |
| --- | --- |
| *Strain and plasmid verification / sequencing* | |
| FC_G3PDH1 | GATCGTTCATGTGGATTTAATTCAAGG |
| RC_G3PDH1 | GACAGAACGTGTCATCACATTCC |
| FC_G3PDH2 | CATTTTCCGTTTCTTGATTATGCGC |
| RC_G3PDH2 | GTTGCTTTGCGGTATTGATCGAC |
| FC_GDH1 | CCCTAAAAACGCTGGAGCAAATTC |
| RC_GDH1 | CATCTATCTGAATCGATTCCATACATGA |
| FC_GDH2 | GCCAATATGAAGAACTTGCTTACAATG |
| RC_GDH2 | CATCACAATACTCCATCGATCATAG |
| FC_LDH2 | CAAGCAGCTTTACCGGATTTTAAAAG |
| RC_LDH2 | GATTTTGTGGAATGATGACATCGTTTC |
| FC_PLF | GGACATGGGCGATTATTTTGCTTTC |
| RC_PLF | GCCGGATCCAAACAGGAACG |
| FC_LDH | GATGTACAACATCAGCAACTACGG |
| RC_LDH | CAATAACCGGAATTCCTATCGCTC |
| FC_ADH | CGAAGAATTGACAAACCGGGAG |
| RC_ADH | TCATACCGCGATCGATTCATCGG |
| FX_NOX | GCAGGATATGATCTGTATTTTTTCAACTC |
| RC_NOX | GAAGAAATCAAACATTTCCTCGAAATCG |
| pLDH_Seq | GTCTGTCATGAAATGGACAAACAATAG |
| ColE_F2 | CCGCCTTTGAGTGAGCTGATA |
| RepB_R1 | CAGCAACTAAAATAAAAATGACGTTATTTC |
| B_ALS_Seq1_R | GAATCACATTTCCCGCTAACGC |
| B_ALS_Seq2 | GCGACGGGATTATTAACAGCGAA |
| B_ALS_Seq3 | GTCGACGATTAATCATATTGAACATGATG |
| pGPAD_Seq | GAAACAACAGCTTTGGCAAATTTTCAAAAG |
| ALD1_Seq | GCGAACATTTGAACATACAGAAGGC |
| ALD3_Seq | GTGGAAGCGTTACTTCATGAATTAATG |
| ALD5_Seq | GATTCGTATTGATGGCACGTTTACG |
| RplS_Seq | CAATCGTTAAAGCGGACGTTTTTGCG |
| Native_NOX_Seq | TCAATTTCTTGATAGGGCGATCAACCATC |
| R_NOX_Seq | CGTGTGTTAGCGAAAAATTTTGATGCGAC |
| Native_BDH_Seq | GGCGGATTCTCAGAATATACCGTCG |
| P_BDH_Seq | CAAGTACAACTTGTGCGACAAGATGG |
| Cas3_sgSeqF | GAAACAACAGCTTTGGCAAATTTTCAAAAG |
| Cas3_sgSeqR | CTTTTGAAAATTTGCCAAAGCTGTTGTTTC |
| FC_Acu | CTTCCATAAACGCCTCAAACAGC |
| RC_Acu | CGTGACCGGGTTAAAGGATG |
| FC_AcoB1 | GATATACCGTTTTTGATCCAGTATTATTG |
| RC_AcoB1 | CATCATCAAAAATGGAAGTGAGTAACAG |
| FC_AcoB2 | GAATATCCGGGAATTGCAAAACATATTAG |
| RC_AcoB2 | CATCCAGCCTAATCCTTGCAATG |
| FC_BDH | GACAAAACTGCAAGCGGTTTGTAC |
| RC_BDH | GAATAAGCGATTTGGAAATGATCGTC |
| FX_NOX | GCAGGATATGATCTGTATTTTTTCAACTC |
| RC_NOX | GAAGAAATCAAACATTTCCTCGAAATCG |
| pLDH_Seq | GTCTGTCATGAAATGGACAAACAATAG |
| ColE_F2 | CCGCCTTTGAGTGAGCTGATA |
| RepB_R1 | CAGCAACTAAAATAAAAATGACGTTATTTC |
| B_ALS_Seq1_R | GAATCACATTTCCCGCTAACGC |
| B_ALS_Seq2 | GCGACGGGATTATTAACAGCGAA |
| B_ALS_Seq3 | GTCGACGATTAATCATATTGAACATGATG |
| pGPAD_Seq | GAAACAACAGCTTTGGCAAATTTTCAAAAG |
| ALD1_Seq | GCGAACATTTGAACATACAGAAGGC |
| ALD3_Seq | GTGGAAGCGTTACTTCATGAATTAATG |
| ALD5_Seq | GATTCGTATTGATGGCACGTTTACG |
| RplS_Seq | CAATCGTTAAAGCGGACGTTTTTGCG |
| Native_NOX_Seq | TCAATTTCTTGATAGGGCGATCAACCATC |
| R_NOX_Seq | CGTGTGTTAGCGAAAAATTTTGATGCGAC |
| Native_BDH_Seq | GGCGGATTCTCAGAATATACCGTCG |
| P_BDH_Seq | CAAGTACAACTTGTGCGACAAGATGG |
| Cas3_sgSeqF | GAAACAACAGCTTTGGCAAATTTTCAAAAG |
| Cas3_sgSeqR | CTTTTGAAAATTTGCCAAAGCTGTTGTTTC |
| AcuA_LHA_F | CATGAGCTAGCGATTCCTAATACCGCAAAATACAGCT |
| AcuA_LHA_R | GTCAGGAAAGATGGTTTAATACATGTGATGTTCCATTAGGTCACCACCTG |
| AcuA_RHA_F | CAGGTGGTGACCTAATGGAACATCACATGTATTAAACCATCTTTCCTGAC |
| AcuA_RHA_R | CATGAGGCGCGCCGAACGCTGGAAATATTCGTATTTCTTTTG |
| AcuA_LHA_F | CATGAGCTAGCGATTCCTAATACCGCAAAATACAGCT |
| AcuBC_LHA_R | GAAATACAAGAATTTGGTAATTTTTCTCATGTTCCATTAGGTCACCACCTG |
| AcuBC_RHA_F | CAGGTGGTGACCTAATGGAACATGAGAAAAATTACCAAATTCTTGTATTTCGC |
| AcuBC_RHA_R | CATGAGGCGCGCCATAGCACATAATCGACAGCGGTTAAT |
| AcuAC_LHA_F | CATGAGCTAGCGATTCCTAATACCGCAAAATACAGCT |
| AcuAC_LHA_R | GTGCATCCTATAGATGTTTTTTAGAAATGATGTTCCATTAGGTCACCACCTG |
| AcuAC_RHA_F | CAGGTGGTGACCTAATGGAACATCATTTCTAAAAAACATCTATAGGATGCACTTA |
| AcuAC_RHA_R | CATGAGGCGCGCCCCAAAAGAAACTGGCAGGATATCG |
| *Cloning Construct* | |
| N_pLDH_F_AvrII | CATCCTAGGGCGGGACGGGGAGCTG |
| N_pLDH_BALS_R | CTTTTTTGTTCTTTCGTCGCTTTCGTCATTGCATTCATCCTCCCTCAATATAATGC |
| N_BALS_LDH_F | GCATTATATTGAGGGAGGATGAATGCAATGACGAAAGCGACGAAAGAACAAAAAAG |
| N_BALS_R_NdeI | CATATCATATGTGCTATCATTCCATACAATATG |
| GPAD_F_NotI | CATTGCGGCCGCCGAAGAAACGAAACAACAGCTTTG |
| GPAD_R_Bot9 | AGTTCTACCTCCTTTATCTAATGCTATACTAATTAACAGCATTTCACAAAAATA |
| E.ALD_F | TAGATAAAGGAGGTAGAACTATGGCGCATGATAAAACGCTTGTC |
| E.ALD_R_XhoI | CATTCTCGAGTTATCCTTCCGCAATTTTAATTTCTTTTTCAATAT |
| Fbpa_F_AvrII | CAGTACCTAGGGAAAGTTGTCACGTTTTCCTTGAGTTA |
| Fpba_R_BALS | TTTTTTGTTCTTTCGTCGCTTTCGTCATGGTAAAATCCTCCTTATGCTTCAAC |
| AlaDH_F_AvrII | CAGTACCTAGGCTATATATCGATATTAAACTTGCCAAATACG |
| AlaDH_R_BALS | TTTTTTGTTCTTTCGTCGCTTTCGTCATCAATGAAATCCTCCTTTGTACTGTAAG |
| BALS_F_Fpba | GTTGAAGCATAAGGAGGATTTTACCATGACGAAAGCGACGAAAGAACAAAAAA |
| BALS_F_AlaDH | CTTACAGTACAAAGGAGGATTTCATTGATGACGAAAGCGACGAAAGAACAAAAAA |
| pLDH_F_AvrII | GAATTCCCAGGCATCAAATAAAACG |
| pLDH_ALS2_R | TCGACTGTGCCTACTCCCATTGCATTCATCCTCCCTCAATATAATG |
| ALS2_native_F | CATTATATTGAGGGAGGATGAATGCAATGGGAGTAGGCACAGTCGA |
| ALS2_R_KpnI | GAATGGTACCTTAAACTAATTGATTAGGTAAAAGTTTTTTCGCAA |
| GPAD_F_AvrII | GAATCCTAGGCGAAGAAACGAAACAACAGCTTTG |
| ALD_R2_R | GTCATAGTAGCACCTCCTTTATATATTATCCTTCCGCAATTTTAATTTCTTTTTCAATAT |
| ALS1_F_R2 | GGATAATATATAAAGGAGGTGCTACTATGACGAAAGCGACGAAAGAACAAAAAA |
| ALS1_R_XhoI | GAATCTCGAGTTATAACGCTTTTGTTTTCATTAATTCGCCAAATT |
| pLDH_F_AvrII | GAATTCCCAGGCATCAAATAAAACG |
| pLDH_ALD_R | ACAAGCGTTTTATCATGCGCCATTGCATTCATCCTCCCTCAATATAATG |
| ALD_Native_F | CATTATATTGAGGGAGGATGAATGCAATGGCGCATGATAAAACGCTTGTC |
| ALD_R2_R | GTCATAGTAGCACCTCCTTTATATATTATCCTTCCGCAATTTTAATTTCTTTTTCAATAT |
| ALS1_F_R2 | GGATAATATATAAAGGAGGTGCTACTATGACGAAAGCGACGAAAGAACAAAAAA |
| ALS1_R_XhoI | GAATCTCGAGTTATAACGCTTTTGTTTTCATTAATTCGCCAAATT |
| GPAD_F_NotI | CATTGCGGCCGCCGAAGAAACGAAACAACAGCTTTG |
| GPAD_R_Bot9 | AGTTCTACCTCCTTTATCTAATGCTATACTAATTAACAGCATTTCACAAAAATA |
| ALD1trunc_Bot9_F | TAGATAAAGGAGGTAGAACTATGATTGGCTTTGATGGACAATTTTATC |
| ALD1_R_XhoI | CATTCTCGAGTTATCCTTCCGCAATTTTAATTTCTTTTTCAATAT |
| ALD3_Bot9_F | TAGATAAAGGAGGTAGAACTATGACGGTAGCGCAACTTATTGATATT |
| ALD3_R_XhoI | CATTCTCGAGTTAGAGCTCTTATTCCGCGCCTT |
| ALD5_Bot9_F | TAGATAAAGGAGGTAGAACTATGAGCTATAACGAAGTCTATCAGTTTA |
| ALD5_R_XhoI | CATTCTCGAGTTAGAGCTCTTATCCTTCGACTTG |
| BDH1_F_PheB | TCTAGATAAGGAGTGATTCGAATGATGAAAGCAGCGCGATGGTACAA |
| RPLS_F_XhoI | CAATCTCGAGAACAATCGTTAAAGCGGACGTTTTTG |
| RPLS_R_PheB | CATTCGAATCACTCCTTATCTAGACAATGCTTTTTCATCATTGCAGCG |
| BDH1_R_NheI | CATTGCTAGCTTATTCTGGTTTGACCAAAATTTTCACTTG |

**Table S5:** Typical metabolite profiles of *P. thermoglucosidasius* NCIMB 11955 WT and *ΔldhΔpfl* (LS100 and TM236) mutants.

| **Strains** | **Pyruvate** | **Lactate** | **Glycerol** | **Formate** | **Acetate** | **Meso-BD** | **RR-BD** | **Acetoin** | **Ethanol** | **Total** | **% pyruvate** |
| --- | --- | --- | --- | --- | --- | --- | --- | --- | --- | --- | --- |
| WT | 0 | 87 | 0 | 0 | 19 | 0 | 0 | 1 | 10 | 117 | 0.00 |
| LS100 | 18 | 6 | 1 | 0 | 12 | 2 | 2 | 0 | 51 | 99 | 0.18 |
| TM236 | 37 | 10 | - | 0 | 15 | - | - | - | 127 | 189 | 0.20 |

Data for TM236 (derived from TM89) are taken from Cripps *et al.* (2009)

**Table S6:** Sequences of synthesized bio-parts used in this study

| **Gene Knockout Cassettes** |
| --- |
| **BDH**  gctagcGAAGCGAATTGCCATTCACTGTTAGGTGGTGCCGTTGTTCTTTTTGCTTGTTCAATAAATGATTCATAAAGCGCAAATCGGACTGGAACTCCACTATTTGTGAGACCAGTCCTTTTATTATTTTTGCAAGCCTCATGTGTCTGCAATTTTTTCAGTATCAGCGGTGTTGAAAATTTTCTTAATAAAATGGATAGATGATAGCGGTAAGGAAGAGGCAAAAACAAAATTGCACCGATCCCCCAGGCTTTTTTGCATGACCGGAGTCCGGCGCCAAAAACGCCGGTATAATGAAGCGCTTTCTATTTATTATATTGTTATATTATTTGTTATTTATAGAAAAGAAATAACTTTAAATGATATAATAATATTGGATATTATAATAATATAAAAAACTGGGAGGGGAAACGAATGAAAGCAGCGAAACCAGAATAAAGAAAAGCAAAAAACCGTTTCAAGGATAAGCCTGCTCTGGATGGATGACTCAGCTTGCCGGCTTTGCCGGCAAGTTGGTCCGTCTTTCAAGAAGATCCTAGTCAGGTGATTAATATGAATATTCATAAATTTGTGATGCCTGAGGTCATTTTTGGGAACGGAGCGATTGAGCATGCGGGGGAAAGCTGTTTGCGGCTTGGGGCAACGAACGTTTTCATTGTCAGCGACCCGGGAGTGATCGAAGCCGGATGGTTAGATGTTGTCATCAGAAGCTGCAAACAGGCGAATTTACAATATACGGTATTTAGCGATGTAACGATGAACCCGAAAGATGTCGAAGTGGAAAAAGGATGCAAAGCTTACATAGAAAATGAATGTGATGCGATCATCGGAATAGGGGGAGGAAGCggcgcgcc |
| **GDH1**  gctagcTAAATTCTTCAAAAGCCCGTTCACCACAGTAACGACAATTCTCCACCAAACGGAACATTTTCTTCTACATTGATCAACTGGCCGTTAATGTGAATTATACCTGTTTTCATTTTCTCGCCGCATGGTGCCCTTGCCAACGGACGCACAGGCTGGCTTGAACAACCCGTCCAAAGCTGCATGAGTACATGCGCGGTTGATGAGTGGATCGGGCGAATTCAATAAACATATTAATTCCAAATCAATTTCAGGAATACTCTATCTATCTCATATTTACAATTTTTGTAATATCGATATTTCTGGATATCGATATTTTTGAATTTATTATGAAATATTTTGTATTAATGCGAAAATAATGTTTTATGGTGATATAATCCTTTGTAGGAAGATAATTCTTTAGGGGGGAGTTCTATGGCCAGAATTGCCGCAATTATTTCAGCGGATATCCTTGGAAGAAAATATAAGTAACCCCCAGCCTCCCCGCGCTAAAAACGCGGGGAGGGGGGCTTGTTCACTCCATTTTCCTTTGCAAAAGAGAGCTTTTGTACTCGTTTGCGGCTTTCCCGCTTTTCTTTCTCGTGTTCCTCTTATTCCTTCAACGTCTTCGATATACATGCCTCGATCACTGTCGTATCCAAATCCGCAGGAAAAACAAGAAGGGGCTACACGATCTTTCTTCATTCCGACAATCCATTCTTCCATAAATTCGTAAAAATCAAATTGGCCATTTATCCTTTGCAACTTCTTTTCACAAAGAGAAAATCCGCCTTGTAAAACAAGAAGATTGCATAAGCTCTATCCGTCCGCGACATAACCATCTTTGAAACATGGTGTTACGTCAAGggcgcgcc |
| **GDH2**  gctagcGTTATCTTCCGTTGTCATAGAGAAAAATATATTGACAATCATTTTTTCAAGAATTATGATTGATGTAAAAATTGAATACGATCACCGCTTATCAAGAGCGACCGAGGGAACAGGCCCTATGACGTCCGGCAACCTCCTTGTAAAATAAAGGAACGGTGCCAAATCCTGCAGAATGATGGCTTTCATTCTGAAAGATAAGTCGTGTGTACATAGATACCCGATGCCTCTTTCTGAATGAAAGAGGCGTTTTTTCTTAGCAGAAAGACGCAAATCTTCTAGTGGTATAAAGTAAATGTTTGCCATCTTCTTTTCATTTCGAAACGATGCAGATCAATAAAACAGGGGAAAGTTCGATGATTGAGGTAAAAAATTTTGTCTATCTTGTCTTTCAAAAGGAGGAATAAAAAGATGGACATGATCTTAGAAGTTTAAACCGCTCTTTGGTTTTCATAATGGCATCATATAAAAATTTGTTAAATAAAAGATAAAGGGGTATTCTTGCTAACTGCCATGTCACGTTCTTTTTCAGGTGATTTTTAACAGTATGCATTGGCATTCTTTAGATCCTTTAGTTTAAACTGTCAACATAAAAGAGGTTGACCCAAAAGTGTCTCACCACACACCAAACTTATAATAATACAGCGTGCCTATTCATATGGCTACGGGATTGGTGCTTTGCTTTAGTTAAGGTCAACCTCTTGGCAGTAACCCCTCTTCATTTAACTTCATTTGAAGAGGGCGAATTTTTTTCGATGGAACTGATATTCCTCTGTATAAAACTCCTCGGGCCTATCTGGAGTATACCGTTGCCAAAATTCCCGGTTTTTGCGGTGCATCTCTggcgcgcc |
| **LDH2**  gctagcGGATTTAGATATGTCTGCGATCATTCAGCCGTTAGAGAAGCGGTGCAATGTGATGGTAACAAAGGAAGAATCATAAGCAGAATTATAAATAAATTCTAAATAAAAAAATTCATTGAAAAATTCGAAAACAGATGGTTTAATAATAAATAATAACACACGCAAAAACGGTTATTCGCTGTTTATCGTTTGCGTGTTTATTTTTATCCATTAAAAGAAACAGTGTTTCTTATTACGAAACAACAAGGAGGTGCAATGCGTGCTGTCTTTGGAGTGGAAAAACAAGTTAACGGAGTTTCTTTCAGAAAAGCAAATACAAATAGATGAACACGGATCGCATCCGCTCGGCAACAGCGGCCATGTGACTGTGTATCCGCAATCGGAAGAAGAAATTTCGAACATTTTGCGCTATGCGAACACCTAGGAGGGATATAAGATGAGTTTGCGAGAAAGTGAAATAAAAAGCGAGCCAGCATGTACAACAATTAGCCTTGGCAACTATCGCTTCAGCGATCCGCCTGATCCAAGCAAGTGGGCTGACTGTGTTCATTGCGGCATGTGTTTGGAGTCGTGTCCGACATACGAACAAACCGGACAAGAACAACATTCGCCGCGCGGGCGCGTGCATTTGATGAAATCGGTGGGAGAAGGGAAGCTGGAGATTACGGAAGATTTGATCGATCCGATTTTTATGTGTTTGGACTGCCGGGCATGCACGACGGCGTGCCCAGCCGATGTGGATGTCGGCGGATTGATTGAAGAAGTACGGGGGCAAATCCGGCAGGCGATTCCGCTAACAGGATGGAAAGCTTTTGTTAACGATTTCTTCTTAAAAGGAGTGTTggcgcgcc |
| **G3PDH1**  gctagcCTGATCGAAAAAACGGAACCTGATTGCATCGAGGTAATCCCTGGAGCGATGCCCCATATTATTCGCGAAGTCAAAGAACGGACGAACAAACCGATTTATGCCGGCGGCTTTATTCGCACTGTCGATGACGTGGAGCGCGCGCTTGAAGCGGGTGCGGTTTCGGTGACCACTTCCAATAAAGACTTGTGGAAACATTATGAGGGGCACTAAGGAGATGCGATGATGCAAGCGGAGCAAGGAAATTTTTTCGCTGCGTCTTACTAGCCAAACAGGCTGTTTTGCGTTATAATGAAACTAAGTTAATAGTTAGTCAGGAGATCTTAGGAGAGACCATGCATGCATGATAAGCAGAAGCTTATCGTGTTTGCAATGGTCTCTTTTTGTTTTTTAATAAAGGGGGAATTTTTCATGGCATCGACAACGTTGTCATAAGTGTGCAATTATTTGCCGCCATCATCATGACGCTCATCGTTGGGCGTCATTTTTTTTGGCAGGATGGATTAAAATAGGTAAAAATGCCGCTGATTAGCATCAATAGGGGGATGAGGATGGGGATTATCTATGATCCAATCATGAGAATATTTCATCTGCAGGCAAATGATATGAGTTATATAATGCAACTTGTCGGACGTGGATATTTAGCCCACTTTTACTGGGGAAAAAGAATCCGGAAAGCGAATGGCTCGCGAAAACTGCGGTTTCTCAGCCGCCCGTTTTCTCCTAATCCCGATCCATCCGCCCGCGCGTTTTCTTTAGATACGCTGCCGCAAGAATACCCGGCATATGGCAACACGGATTTTCGCGCGCCGGCGTATCAAGTGCAATTAGAAAACGGGTCGAggcgcgcc |
| **G3PDH2**  gctagcAAGATTTATTATATGACGCAAGTAGCGGTAAAACCGCCAACTTTTGTCGCGTTTGTCAACGACCCGGAGCTGATGCACTTTTCTTACGAGCGCTTTTTGGAAAATCGCATCCGCGATGCGTTCGGCTTTGAAGGGACACCGATAACAATCATTGCCAGACCGAGAAAATAACAAAAAGCGCAGGACGCCTCCCATCCGCCGAAAATCTGCCCGCGTTTCGAAAACTCGGCAAAGAGACTGCGGGAAGGTTCAGGCTAAACAAAATTTTGTTTTGTATGGGTCGCCTGGCAGTAAACGCAATGTGCTGCCGACGCAGAAACCGAAGCGCCGCGGGCGGATGGCGCCCGGAGCCAGACAAAAGCAGAACGCGAAGCGACAGGCAATTTATAAATAATTAAGGAAGTGAGAATGGTGGAACAACGGGAAAAATGAGGAAAATAGGCGTTCGATGATACAAGGCGTTTGCGCTTCGCATACAATACTACGAACAATTATAGACTTGAGCTTCCGGACCAAGGGTGTTGTTGTTCAACTGAAGTAAAGAAGAGCCCCGCTTTACTTCAGTTTTTTATTCGTGCATAAAGGTCGCGCCTGCGTGAAACAAAATATCGTTATTTGTTATAATAGTGTCGAAAAAAGGAGGAAAAGGGATGTCACCGGCGTTGGCAAAAATGTGGATTGCCATTGCGTCCATGGTATTCATGTTTATCTCGGTATTTTCGATTTATATCAGCCGTTATAAAGTAAAAAATAAGATTATTCGCTTTATTTTGGCATTGATCGCCTATGTTTTTATGATTTTAGCAGGAATTATCATTATTTTTGTCGTGTTTAGCGGCCggcgcgcc |
| **AcoB1**  GGATCCCGTAATTGCCGTATACGAGGGTTTTAGAGCTGTGTTGTTTGTTAAAACAACACAGCGAGTTAAAATAAGGCTTAGTCCGTACTCAACTTGAAAAGGTGGCACCGATTCGGTGTTTTTGTCGACGAATTCGCGGCCGCACTAGTCCAGGCATCAAATAAAACGAAAGGCTCAGTCGAAAGACTGGGCCTTTCGTTTTATCTGTTGTTTGTCGGTGAACGCTCTCCTGAGTAGGACAAATCCGCCGGGAGCGGATTTGAACGTTGCGAAGCAACGGCCCGGAGGGTGGCGGGCAGGACGCCCGCCATAAACTGCCAGGCATCAAATTAAGCAGAAGGCCATCCTGACGGATGGCCTTTTGCTAGCCTAATGAAGTGATCCAACTGTTTATGACGTACCATTGGCCAGGAAATATTCGCGAATTATTTAACGTGTTGGAGCGGATTCGGATTGAATATGGCGATCATATTCCTTCGATTCCAGAGCTTAAATCGATGTTCATTGGATGGGAAAATCAAGGAGAACACAGAAAGTCAGAGCAAAAAACTTTATCCTATCGGGAACAGATTGAAAAAAATCATATGATGGAGATGCTGGAAAAAACAAAAGGGGATATTGCAAGGGCAGCAGCAGAGTTGAATATTCCCCGCAGTACATTTTACCGAAAATTAAAAAAATATCATTTGATATGAGACAAAATGAGAAAGAATGAGAAAAAATGGGATTATTCCGAATTTATTTTATTGAAATCACTTACAAAAAAGGGTTTGTAAGCGTTTTTTTGTTGGCATATTATTTGCATAGTTTGAAAGTGAAAAGGTTTCATGATCATTATGAGCAGTTGTAAAGGAGGAAAAGGAAAGCATGACAGTGTCTTACTAATTGTCAAAGTTAGGAGGGAATAATCAACTATGACAAGAACCATTTCTTTTTCAGCAGCAATTAATGAAGCGATGAAGCTCGCGATGCGCAAAGATGAAAATGTTATTTTGTTGGGCGAAGATGTAGCTGGCGGTGCAACTGTCGATCATTTGCAAGATGAGGAAGCTTGGGGAGGTGTAATGGGCGTCACCAAAGGCCTCGTCCAAGAATTTGGCAGAGAAAGAGTATTAGATACACCAATTGCGGAAGCTGGATATATTGGGGCAGCGGTTACCGCAGCGGCGACGGGATTAAGGCCGATTGCCGAATTGATGTTTAACGATTTTATCGGCAGCTGTTTAGATGAAGTTATGAACCAAGCAGCTAAACTTCGCTATATGTTTGGCGGAAAAGCGAAAGTGCCTTTAACTATACGGACGATGCATGGTGCCGGATTTCGCGCGGCCGCGCAGCATTCACAAAGCCTATACGCGATTTTCACCCATATACCAGGGCGCGCCGATGGTTTTGAACTTGTTCTTTCTTATCTTGATACATATAGAAATAACGTCATTTTTATTTTAGTTGCTGAAAGGTGCGTTGAAGTGTTGGTATGTATGTGTTTTAAAGTATTGAAAACCCTTAAAATTGGTTGCACAGAAAAACCCCATCTGTTAAAGTTATAAGTGACTAAACAAATAACTAAATAGATGGGGGTTTCTTTTAA |
| **AcoB2**  GGATCCGCCGGCATACCCCATGCGCTGTTTTAGAGCTGTGTTGTTTGTTAAAACAACACAGCGAGTTAAAATAAGGCTTAGTCCGTACTCAACTTGAAAAGGTGGCACCGATTCGGTGTTTTTGTCGACGAATTCGCGGCCGCACTAGTCCAGGCATCAAATAAAACGAAAGGCTCAGTCGAAAGACTGGGCCTTTCGTTTTATCTGTTGTTTGTCGGTGAACGCTCTCCTGAGTAGGACAAATCCGCCGGGAGCGGATTTGAACGTTGCGAAGCAACGGCCCGGAGGGTGGCGGGCAGGACGCCCGCCATAAACTGCCAGGCATCAAATTAAGCAGAAGGCCATCCTGACGGATGGCCTTTTGCTAGCAACGATTTGCCGGAAGAAATGTTTCAAATGTTTCCAAGTTTGAAGAAGAAAGAAGTGCCGGTGCATAGAGACGATATTAAAAAACAGTCCATCATTCAGGCATTACGTTTATGCAACGGGAATATTTCGAAAGCGGCTAAGCATTTAGGGATTTCCAGAAGCACTTTTTATCGGCAAATGCGAAAATTTAACATCGCTTATTAGCTTGGAGTAATATTGTTTCATTTTGTATCAAATTGTATCAATATGTAGCAACAAAGTGTTCCAAAATGGGACATTTTGTTGCTTTTTTTCTATAAAGCGGATGATCTCATAAAGAAAATTTTTAGCGGAATGAGAATGGTTTGCGGTAAATTCCACCATGCGCCCTGCTGCATTGGCACGTTTTTTGCTTTTTAAATATAAATGAAAACGCTTTATTTTAAAATAAAGGAGGAGAGCAAGTTGGTGATTTCGATTGACCATCTTATTTGGATGTATGAAACAATGTACAAAATCCGTTTTGTTTAAAACACGAGAGAAAGGGGTCATCGAAATGCAAAAAACAAAGCGTCGTTTATTAACAGGAAATAAAGCGATTGCCGAAGCGATCCGCCAAGAAATGGAACGAGATCCGAGCGTGTTTGTCATGGGAGAGGATGTCGGTGTATACGGAGGCATATTCGGGGCGACGGAAGGATTATTTCAACAATTCGGTCCTGAAAGGGTGATGGATACACCGATTTCCGAAACGGCATTTATTGGGGCGGCGATTGGCGCCGCTGCAGAAGGGATGCGGCCGATTGTTGAATTAATGTTCGTTGATTTCTTCGGCGTTTGCATGGACCAAATTTATAACCATATGGCCAAAATTCCATATATGTCGGGAGGACGGGTGAAGCTGCCGATCGTGTTAATGACCGCTGTTGGCGGGGGATACAGCGATGCCGCCCAGCATTCGCAAACATTATATGCCACTTTTGCCCATCTTCCGGGAATGAAAGTGGTAGCGCCATCCACTCCGGCGCGCC |
| **AcuA**  GGATCCTCAATAATCAGCGTTCCCGTTTTAGAGCTGTGTTGTTTGTTAAAACAACACAGCGAGTTAAAATAAGGCTTAGTCCGTACTCAACTTGAAAAGGTGGCACCGATTCGGTGTTTTTGTCGACGAATTCGCGGCCGCACTAGTCCAGGCATCAAATAAAACGAAAGGCTCAGTCGAAAGACTGGGCCTTTCGTTTTATCTGTTGTTTGTCGGTGAACGCTCTCCTGAGTAGGACAAATCCGCCGGGAGCGGATTTGAACGTTGCGAAGCAACGGCCCGGAGGGTGGCGGGCAGGACGCCCGCCATAAACTGCCAGGCATCAAATTAAGCAGAAGGCCATCCTGACGGATGGCCTTTTGCTAGCGATTCCTAATACCGCAAAATACAGCTCCGGCGAACGCGGCATAAAAACAAATACACGGTCCCCTTTTTCGATCTCTGCTACTTCTTTCAATACGTTTGCCACTTTGTTGGACAATTCTTTCATTTCTTTAAACGTATACTTTTCATTTCTCGACGCATCGCGGTAATAAAGCGCCACTTTATTTTTTCGGAACGTTTCCACATGGCGGTCAATCGCCTCATATGCCATATTGACCCGTCCTGTTTCAAACCAGGAAAATTCCTTTTCTACTTCCTTCCAGTCAAAATTTTTATATACCTCTTCATAATTTTTTAAGTTATAATCCCCTTGTACGACCGGTAGCACTTCTGTTTTCATTCTCCAAATCCCCCTCGTGTAAGTGTTTACAACCCTATTATAGTATATATCTTATAAATTCTCAATTTTTTAAAAATTTAACTATTAAAATTTTTTTCAAATTTACTTATCGTATTTTCATGTATAATGAAAACAGAAGAAGAAATTCCAGGTGGTGACCTAATGGAACATCACATGTATTAAACCATCTTTCCTGACAATAAAGAGGTGAAGAAGAATGCTCGTCGAGCAAATCATGAAAACACCCGTCATTACGCTGCAACCAACAAACACGATTGCCGAAGCAATCCAGTTAGTGAGGCAACGCCGCATTCGCCATATTCCGATTGTGGATGGCGACGATCATGTCGTCGGCATTGTGACCGATCGTGATATCCGTGATGCAAGCCCTTCCATCTTCCATTTTCATGAACATCTCGAAGATTTGCAAAAACCAATCAGCACGATTATGAAGACAGAGGTCATCGTCGGGCATCCGCTTGATTTCGTTGAAGAAGTGGCAGCACTGTTTTATGAACATAAAATTAGCTGTTTGCCTATTGTTAAAGATCGCAAACTTGTCGGCATCGTAACAGAGACAGATTTATTATATACGCTTATCCAGCTGACTGGTGCGCATCAGCCAGGCACGCAAATCGAAATAAAAGTTCTAAATGAAAGCGGGATGCTAAGCAAAGCAGCCGCGATTATCGCCAAAAGAAATACGAATATTTCCAGCGTTCGGCGCGCC |
| **AcuB**  GGATCCTATACGCTTATCCAGCTGACGTTTTAGAGCTGTGTTGTTTGTTAAAACAACACAGCGAGTTAAAATAAGGCTTAGTCCGTACTCAACTTGAAAAGGTGGCACCGATTCGGTGTTTTTGTCGACGAATTCGCGGCCGCACTAGTCCAGGCATCAAATAAAACGAAAGGCTCAGTCGAAAGACTGGGCCTTTCGTTTTATCTGTTGTTTGTCGGTGAACGCTCTCCTGAGTAGGACAAATCCGCCGGGAGCGGATTTGAACGTTGCGAAGCAACGGCCCGGAGGGTGGCGGGCAGGACGCCCGCCATAAACTGCCAGGCATCAAATTAAGCAGAAGGCCATCCTGACGGATGGCCTTTTGGCTAGCCGGAAGGAAGAATCATCATCGCCCGCCATCATCAAACAATCGTCGGTTATGTTACATTTCTTTACCCTGATCCGCTGGAACGATGGTCGGAAGGAAATATGGAAAATTTAATTGAATTGGGTGCGATTGAAGTCATTCCGGAGTTTCGCGGCTATGGCGTGGGAAAAAACTTGCTGATTGTATCCATGATGGATGATGCGATGGAAGATTACATTATTATTACGACAGAATATTACTGGCATTGGGATTTAAAAGGAACAGGATTAAACGTATGGGAGTACAGAAAAGTAATGGAAAAAATGATGAACGCTGGCGGGCTTGTTTGGTATGCAACAGATGATCCCGAAATTTGCTCTCATCCAGCTAACTGCCTGATGGTCCGCATCGGGAAACGGGTCGATCAAGAATCCATCCAAAAATTTGACCGCCTTCGCTTCATGAACCGGCACATGTATTAAACCATCTTTCCTGACAATAAAGAGGTGAAGAAGAATGCTCGTCGTGTTATGGCCGAATTTGCCGGGGGTTTCGTCATGAAAAAAGATTGCGTATTTATTTACAGCGAAGATTTCCTCCGATATAAATTTCATGACGCGCACCCGTTTAACCAGCTGCGTGTCAAGCTGACATACGACTTATTGCGCGCAATGAACGCACTGGAAGATGAGCAGATCATCGAACCAAGAATCGCGACGGACGAGGAACTCGCCTTGATTCATGATCAAACATATATTGAAGCGGTCAAAGCGGCCGGAAAAGGGCAGCTGCCTGAGCATACCGCCTTAAACTACGGGCTGGGAACGGAAGATACGCCAATTTTCCCAAATATGCATGAAGCAAGCGCATTGTTGGTGGGAAGCACATTAACCGCTGTCGATTATGTGCTATCCGGAAAAGCAAAGCATGCGCTAAGTTTGGGCGGCGGGCTTCACCACGGCTTCCGCGGCAAAGCATCCGGATTTTGCGTTTACAATGATAGCGCTGTCGCCATTAAATACATTCGGCGCGCC |
| **AcuC**  GGATCCGATGTGATCTTGACGCAAAAGTTTTAGAGCTGTGTTGTTTGTTAAAACAACACAGCGAGTTAAAATAAGGCTTAGTCCGTACTCAACTTGAAAAGGTGGCACCGATTCGGTGTTTTTGTCGACGAATTCGCGGCCGCACTAGTCCAGGCATCAAATAAAACGAAAGGCTCAGTCGAAAGACTGGGCCTTTCGTTTTATCTGTTGTTTGTCGGTGAACGCTCTCCTGAGTAGGACAAATCCGCCGGGAGCGGATTTGAACGTTGCGAAGCAACGGCCCGGAGGGTGGCGGGCAGGACGCCCGCCATAAACTGCCAGGCATCAAATTAAGCAGAAGGCCATCCTGACGGATGGCCTTTTGCTAGCTCGTGATATCCGTGATGCAAGCCCTTCCATCTTCCATTTTCATGAACATCTCGAAGATTTGCAAAAACCAATCAGCACGATTATGAAGACAGAGGTCATCGTCGGGCATCCGCTTGATTTCGTTGAAGAAGTGGCAGCACTGTTTTATGAACATAAAATTAGCTGTTTGCCTATTGTTAAAGATCGCAAACTTGTCGGCATCGTAACAGAGACAGATTTATTATATACGCTTATCCAGCTGACTGGTGCGCATCAGCCAGGCACGCAAATCGAAATAAAAGTTCTAAATGAAAGCGGGATGCTAAGCAAAGCAGCCGCGATTATCGCCAAAAGAAATACGAATATTTCCAGCGTTCTTTTATATCCGGCTGAAGAGAAAAATTACCAAATTCTTGTATTTCGCGTGCAAACAATGAATCCGATCGGAATCATCAATGATTTAAAAAATGCCGGCTATACCGTGTTATGGCCGAATTTGCCGGGGGTTTCGTCATGAAAAAACATTTCTAAAAAACATCTATAGGATGCACTTACCTACGAGTGTAGTGAAACACGAAAAAAGGGGCTTACCGAAACGGTAAGCCCCTTTTTTCGTTCGTTATTTCGTTGATTCGCGATATTCAATGCGATGCGGCAAAACGACAATATGGTTTTCCACATTTTCTTTATTCATATACTTGGTCAATAAACGCATCGCAACCGCACCGATATCATACATCGGCTGCATCACGGTCGTTAAACGAGGGCGAACCATCGTTGCAAGCCGCGTATTGTCAAAGCCGATCACTTCTAATTGGTCAGGAATGCGAACGCCATGGTCTTGCGCGCTATGGATCACACCAAGCGCCATCTCGTCCGTTCCGGCAAAAATGGCGGTTGGCTTTTCCGCTAACTCCGCTATTTTTTCATATGCCTCAATACCAGAATCGTACGAATTGTCTCCTTCGATGACAAGCTCTTCATCATACGTTAATCCGTGCGTTTCTAAAGCGCGGCGATATCGGCGCGCC |
| **AcuAC**  GGATCCTCAATAATCAGCGTTCCCGTTTTAGAGCTGTGTTGTTTGTTAAAACAACACAGCGAGTTAAAATAAGGCTTAGTCCGTACTCAACTTGAAAAGGTGGCACCGATTCGGTGTTTTTGTCGACGAATTCGCGGCCGCACTAGTCCAGGCATCAAATAAAACGAAAGGCTCAGTCGAAAGACTGGGCCTTTCGTTTTATCTGTTGTTTGTCGGTGAACGCTCTCCTGAGTAGGACAAATCCGCCGGGAGCGGATTTGAACGTTGCGAAGCAACGGCCCGGAGGGTGGCGGGCAGGACGCCCGCCATAAACTGCCAGGCATCAAATTAAGCAGAAGGCCATCCTGACGGATGGCCTTTTGCTAGCGATTCCTAATACCGCAAAATACAGCTCCGGCGAACGCGGCATAAAAACAAATACACGGTCCCCTTTTTCGATCTCTGCTACTTCTTTCAATACGTTTGCCACTTTGTTGGACAATTCTTTCATTTCTTTAAACGTATACTTTTCATTTCTCGACGCATCGCGGTAATAAAGCGCCACTTTATTTTTTCGGAACGTTTCCACATGGCGGTCAATCGCCTCATATGCCATATTGACCCGTCCTGTTTCAAACCAGGAAAATTCCTTTTCTACTTCCTTCCAGTCAAAATTTTTATATACCTCTTCATAATTTTTTAAGTTATAATCCCCTTGTACGACCGGTAGCACTTCTGTTTTCATTCTCCAAATCCCCCTCGTGTAAGTGTTTACAACCCTATTATAGTATATATCTTATAAATTCTCAATTTTTTAAAAATTTAACTATTAAAATTTTTTTCAAATTTACTTATCGTATTTTCATGTATAATGAAAACAGAAGAAGAAATTCCAGGTGGTGACCTAATGGAACATCATTTCTAAAAAACATCTATAGGATGCACTTACCTACGAGTGTAGTGAAACACGAAAAAAGGGGCTTACCGAAACGGTAAGCCCCTTTTTTCGTTCGTTATTTCGTTGATTCGCGATATTCAATGCGATGCGGCAAAACGACAATATGGTTTTCCACATTTTCTTTATTCATATACTTGGTCAATAAACGCATCGCAACCGCACCGATATCATACATCGGCTGCATCACGGTCGTTAAACGAGGGCGAACCATCGTTGCAAGCCGCGTATTGTCAAAGCCGATCACTTCTAATTGGTCAGGAATGCGAACGCCATGGTCTTGCGCGCTATGGATCACACCAAGCGCCATCTCGTCCGTTCCGGCAAAAATGGCGGTTGGCTTTTCCGCTAACTCCGCTATTTTTTCATATGCCTCAATACCAGAATCGTACGAATTGTCTCCTTCGATGACAAGCTCTTCATCATACGTTAATCCGTGCGTTTCTAAAGCGCGGCGATATCCTGCCAGTTTCTTTTGGGGCGCGCC |
| **Synthetic Genes** |
| ***Bacillus Subtilis* Acetolactate synthase (ALS1)**  ATGACGAAAGCGACGAAAGAACAAAAAAGCCTTGTCAAAAATCGCGGAGCGGAATTAGTAGTAGATTGTTTAGTAGAACAAGGCGTTACACATGTGTTTGGCATTCCGGGAGCGAAAATTGATGCGGTATTTGATGCGTTACAAGATAAAGGACCGGAAATTATTGTTGCGCGCCATGAACAAAATGCGGCGTTTATGGCGCAAGCGGTGGGCCGCTTAACGGGAAAACCGGGAGTGGTTTTAGTTACGAGCGGACCGGGCGCGTCGAATTTAGCGACGGGATTATTAACAGCGAATACAGAAGGAGATCCGGTAGTGGCGTTAGCGGGAAATGTGATTCGCGCGGATCGCTTAAAACGCACACATCAAAGCCTTGATAATGCGGCGTTATTTCAACCGATTACGAAATATTCGGTCGAAGTACAAGATGTTAAAAATATTCCGGAAGCGGTCACAAATGCGTTTCGCATTGCGAGCGCGGGCCAAGCGGGAGCGGCGTTTGTTAGCTTTCCGCAAGATGTTGTCAATGAAGTGACAAATACGAAAAATGTCCGCGCGGTTGCGGCGCCGAAATTAGGACCGGCGGCGGATGATGCGATTTCGGCGGCGATTGCGAAAATTCAAACGGCGAAATTACCGGTGGTTTTAGTGGGCATGAAAGGAGGCCGCCCGGAAGCGATTAAAGCGGTACGCAAATTATTAAAAAAAGTGCAATTACCGTTTGTTGAAACATATCAAGCGGCGGGAACATTAAGCCGCGATTTAGAAGATCAATATTTTGGCCGCATTGGATTATTTCGCAATCAACCGGGCGATTTATTATTAGAACAAGCGGATGTCGTGTTAACGATTGGCTATGATCCGATTGAATATGATCCGAAATTTTGGAATATTAATGGAGATCGCACGATTATTCATTTAGATGAAATTATTGCGGATATTGATCATGCGTATCAACCGGATTTAGAATTAATTGGCGATATTCCGTCGACGATTAATCATATTGAACATGATGCGGTTAAAGTGGAATTTGCGGAACGCGAACAAAAAATTTTAAGCGATTTAAAACAATATATGCATGAAGGCGAACAAGTTCCGGCGGATTGGAAATCGGATCGCGCGCATCCGTTAGAAATTGTGAAAGAATTACGCAATGCGGTGGATGATCATGTTACGGTAACATGCGATATTGGATCGCATGCGATTTGGATGTCGCGCTATTTTCGCAGCTATGAACCGTTAACGTTAATGATTAGCAATGGAATGCAAACGTTAGGCGTAGCGTTACCGTGGGCGATTGGCGCGTCGTTAGTTAAACCGGGAGAAAAAGTGGTATCGGTAAGCGGCGATGGAGGCTTTTTATTTTCGGCGATGGAATTAGAAACAGCGGTTCGCTTAAAAGCGCCGATTGTGCATATTGTATGGAATGATAGCACATATGATATGGTAGCGTTTCAACAATTAAAAAAATATAATCGCACAAGCGCGGTCGATTTTGGCAATATTGATATTGTTAAATATGCGGAATCGTTTGGCGCGACAGGCTTACGCGTCGAATCGCCGGATCAATTAGCGGATGTCTTACGCCAAGGCATGAATGCGGAAGGCCCGGTGATTATTGATGTCCCGGTCGATTATTCGGATAATATTAATTTAGCGTCGGATAAATTACCGAAAGAATTTGGCGAATTAATGAAAACAAAAGCGTTATAA |
| ***Bacillus Coagluan* Acetolactate synthase (ALS2)**  ATGGGAGTAGGCACAGTCGAGAAAAAGAATAGCAATCCTACGAATACGACGGAAAAAACAGCGGCGGATCTTGTGGTAGATTGCCTTGAAAAACAAGAAGTGCCGTATGTGTTTGGCATTCCGGGAGCGAAAATTGATGCGGTGTTTGATGTACTTAAAGAACGGGGACCGGAACTTATTGTGTGTAGACATGAACAAAATGCGGCGTTTATGGCGGCGGCGATTGGCCGCCTTACAGGCAAACCTGGGGTCTGCTTAGTCACATCGGGCCCAGGCGCGTCCAATCTTGCGACAGGACTTGCGACAGCGAATACAGAATGTGATCCTGTGGTCGCGATTGCGGGCAATGTCCCTCGTGCGGATCGCTTGAAGAAAACTCATCAAAGTATGGATAATGTCTCGTTGTTTCAACCTATTACGAAATATGCGGCGGAAGTGGTGCATCCTGATACAGTGCCGGAAGTAATGACGAATGCGTTTCGCTCCGCGGCGTCCGCGCAAGCGGGAGCGGCGTTTATTAGCTTTCCACAAGATGTACTTAAAGAACCTGCGTCGGTTAAAGCGTTGGGCCCTCTTAAATCCCCTAAACTTGGCAAAGCGAACGAAGAAGCGGTCAAAGAAGCGGTCAAAGCGATTCAACATGCGAAATTACCAGTCATTTTGGTAGGCATGAGAGCGAGCAGACCGGAAGTGGTCAAAGCGGTACGCTCCCTTCTTAAAAAGATTGCGCTTCCAGTAGTGGAAACGTTTCAAGCGGCGGGCCTTATTAGCCGTGATCTTGAAGATCGCTTCTTTGGACGCATTGGCTTGTTTCGTAATCAACCGGGAGATATTCTTCTTGAACATGCGGATGTAGTGTTAGCGATTGGCTATGATTCGGTAGAATATGATCCTAAATTTTGGAACTCCGAAGGTGAACGCAAGATTATTCATCTCGATGAAATTCGTGCGGATATTGATCATGATTATCAACCTGAAATTGAACTTGTGGGGGATATTTCCGCGTCCGTCGATAGCATTAAAGAACAACTTGCGCGCCTTAATATGAATGGCAAATCGATGGAACTTCTTGAACGGTTACGCAATCAACTTAATTTGAGAGATGAACCGTCCGAAAAAGCGGATAAAAATCTTGTACATCCTTTACAGTTTATTCATGATCTTCGCTCCCTTATTGATGATCATGTGACAGTGACGTGTGATGTGGGCAGCCATTATATTTGGATGGCACGCCATTTTCGGGTGTATGAACCTAATCGGTTGTTGTTTAGCAATGGTATGCAAACGTTGGGAGTCGCGTTACCGTGGGCGATTGCGGCGACGTTAGTTAATCCTGGAGAAAAAGTAGTCTCCATTTCGGGAGATGGAGGCTTTTTGTTTTCCGCGATGGAATTAGAAACGGCGGTCAGACTTAAATCGCCGTTGGTTCATATTGTCTGGAGAGATGGAACTTATGATATGGTCGCGTTTCAACAACAGATGAAATATGGCCGCACATCGGGCGCGGATTTTGGACCTGTCGATATTGTCAAACATGCGGAATCGTATGGAGCGAAAGGCTTGAGAGTTAATAGCCCAGATGAATTAGTCTCGGTGCTTAAAGAAGCGTTAGATTCCGAAGGACCTGTCGTGGTAGATGTACCAGTCGATTATTCCGATAACCTGGAACTTGCGAAAAAACTTTTACCTAATCAATTAGTTTAA |
| ***Exiguobacterium acetylicum* acetolactate decarboxylase (ALD1)**  ATGGCGCATGATAAAACGCTTGTCCAAATTTCCACAATGATGGCGTTGTTAGATGGAGTCTTTGAATCCGAAGTCACCTATGCCTCCGTGTTAGATCAACGGGATTTTGGCATTGGCACGTTTGATCACCTGGATGGAGAAATGATTGGCTTTGATGGACAATTTTATCAACTTCGCAGCGATGGATCGGCGAGACCGTTGCAACCTGAAACGACGACGCCTTTTGCGACACTTACGCGCTTTGAACCAGAACAAACACTTACAGTAACGGAAGAAATGAGCAAAGCGACATTTGAACATTGGTTAAACGAACAACTTCCTACGATTAATAGCTTTTATGCGATTCGTATTGATGGCACGTTTACGGAAGTGCAAACACGCACAGTAGCGCGTCAAGAAAAACCGTTTGTACCTATTACAGAAGCGGTCGCGAGCCAATCCGCGCGAACATTTGAACATACAGAAGGCACACTTGCGGGCTATTATACACCTCGGTTTGGTCACGGAATTGCGGTAGCGGGCTATCATCTTCATTTTATTGATGCGGCGAGAGAAGGGGGAGGCCATGTGTTTGATTATACAGTTAAAAATGTGACAGTGACGTTTGAAGAAAAACCTCAATTAGACCTTCGCCTTCCTACGACGGAAGCGTATCGCTCGGCGGATCTTGAATCCCATGATATTGAAAAAGAAATTAAAATTGCGGAAGGATAA |
| ***Bacillus coagluan* acetolactate decarboxylase (ALD2)**  ATGAGAACAGCGGCGGAAAATCAAAATACAGAACAAATTCTTACGAGCCGCACAGATGAAGTCTATCAACTCTCCACAATGACGTCCTTGTTAGATGGAGTCTATGAATCCGATAAAACGTTTGCGGAACTTAAAAAATTTGGCGATTTTGGCATTGGCACGTTTAATCATCTTGATGGAGAACTTATTGCGTTTGATAATGCGTTTTATCAACTTAAAGATGGCACGGCGAAACGGGTGCAACCTGAAGATAAATCGCCGTTTTGCTCGTTAGCGCATTTTTCCGAAGATATTACATATACGGCGGAAGGACCACTTGCGAAACCTGAATTAGAGGATCTTATTAAGGATCTTGTCCGCTCCGAAAATTTGTTTTATGCGATTCGGGTCGATGGAGTTTTCAAGAAGATGAATACTCGAACAGTGAGCTATCAAGAAAAACCAGTACCTATGACGGAAGCGGTCAAATCCCAACCGGTGTATAGCTTTGAAAATACGAAAGGCACGTTAGCGGGCTTTTGGACACCAATGTTTGCGCAAGGCATTGCGGTAGCGGGCTTTCATCTTCATTTTATTGATGATAAACGTACCGGGGGAGGCCATGTGTTTGATTATGTGTTGGATTATGGCACGATTCGTATTAGCAAGAAAACTCACATGCATCTTGAACTTCCTGAAACGGATGCGTTTCTTAATGCGAATCTTTCCCGCGCGAACTTGGCGGAAGAATTGGAAAAAACAGAAGGCTAA |
| ***Bacillus cereus* acetolactate decarboxylase (ALD3)**  ATGACGGTAGCGCAACTTATTGATATTGATGCGAAGAAAACAAAAACGTCCAATGAGGTCTATCAAACATCCACAATGTTAGCGTTGTTAGATGGCATCTATGATGGAGTAATTTCCTTTGAGGACCTTAAAAAACATGGAGATTTTGGCATTGGCACGTTTGATCAATTAGATGGCGAAATGATTGCGTTTGATAACGAGTTTTATCATCTTCGCTCCGATGGCTCCGCGGAAAAAGTCGAACCGGAAGAAACAACGCCGTTTGCGACGGTGACGTTCTTTGAAAAAGAAATGTCGTATACGGTCGAACGCCCTATGAATCGGGAAGAAGTGGAAGCGTTACTTCATGAATTAATGCCGAGCAAAAATTTGTTTTATGGAATCCGTATGGATGGCACATTTCGCGAAGTGAGAACGAGAACAGTACCTAGACAAGAAAAACCATATACGCCTCTTGTAGAAGTCACGAAATCCCAACCTATTTTTAGCTTTGAAAATACAGAAGGCACACTTGCGGGCTTTTGGACACCTGATTATGCGCAAGGCATTGGAGTCGCGGGCTTTCATCTTCATTATATTGATGATGAACGCTCGGGTGGGGGCCATGTGTTTGATTATGTCATTGAAAATTGTACGATTCAAATTTGCCAAAAAGCGCACATGCATCTTGCGCTTCCAGAAACAGCGGATTTTATGGCGGCGGCGTTAAGCAGAGAAAATCTTGAAGATAATATTGCGACAGCGGAAGGCGCGGAATAA |
| ***Pelotomaculum thermopropionicum* acetolactate decarboxylase (ALD4)**  ATGACGAGACATTTGTATCATGTAATTGCGGCGATGTTGTTAGCGGCGTTGCTTGCGTTGGCGGGCTGTGCGCAAGCGGGCAAATCCGAAAAACTTCGTGAAGCGGGACCACCTGAAGATACAGTCTTTCAAGTCTCGACAATTAATGCGTTATTACAAGGCTTGTATGATGGAGAAGTGACGTGTGGCGAACTTAAAAAACATGGAGATTTGGGAGTGGGCACGTTTGATGGCCTTGATGGAGAAATGGTGGTCGTCGATGGAATTATTCTTCAAGTTAAAGCGGATGGCAAAGTACTTCCTGCGCCAGATGGAGAAAAAACACCGTTTGCGGCGGTAACGTTCTTTAGCTCCGATCGCACACAACAAGTTAAAGAATTGGCGGATTATAGCCATCTTCAACGGTTGTTAGATGGCCTTATTGCGAATCGCAATATGTTTTATGCGATTCGCATTGATGGCACATTTCCATACGTCAAAACGCGCTCGGTTCCTGAACAAGCGAAACCGTATCCACCTTTAGCGGAAGTCACGAAAAATCAACCAGTGTTTGAAATGCGTAATGTCAGAGGCTCGGTAGTAGGCTTTTATTGCCCTCCATATATTGAAGGACTTAATGTCCCAGGCTATCATCTTCATTTTGTGACAGAAGATAGACGCCAAGGTGGCCATCTTCTTGAATGCAGCTTACAAGAAGGAACACTTCAGATGGATCAAACGAGAGGCTTTTATATGACATTACCGGCGGGCTCAGATTTTGCGAAAGCGGATCTTACGACAGGACGTACGGAAGAACTTAAAAAAGCGGAATCGTAA |
| ***Chaetomium thermophilum* acetolactate decarboxylase (ALD5)**  ATGAGCTATAACGAAGTCTATCAGTTTAGCATTGTATCCGCGCTTATGGATGGAGTCGCGTCGCATGGAGTGCCGATTAGCCGTATTCTTGCGCATGGAGATCATGGCCTTGGCACGTTTCGGTATATGGATGGAGAAATGATTGTACTTGATGGCCAATGCTATGCGATGAAACATGATGGCTCGGTTATTCATATTGCGGACCCGTCCGCGGTACTTACGCCGTTTGCGACAGTCACACGCTTTAAACCTACGATTGAAACAAGAGCGGCGGTCAAAGGCAAACAAGATTTTACACGCCTTCTTAATGGCTTGTTTCCTAAAGCGCGTAATCATTTTCTTGTGGTTCGTATTGATGGCGTGTTTGCGAAAGTAGTAGTGAGAACAGCGGGAGGACAAATTAGACCTAGAGAAGGCATGGTCGATGTATGCAGCCGCCAAACGACACATAATTTTGAAAAAGTAAAGGGGGCGGTGGTGGGCTTTCGCTGCCCTGAATATATTATGGGCGTCAATGTCGCGGGCGATCATTTTCATTTTATTGCGGAAGATAGACAACGGGGAGGCCATATTCTTGAATTTGAAACGGATGGCGAAGTTAATGTCAAAGCGGCGCAAATTGCGACGTTTCACATGGATCTTCCTACAGAAGATGATGAGTTCAACGAAGCGGAACTTAAACTTCAAGCGCAAGGCATTAAACAAGTCGAAGGATAA |
| ***Tribulus Terrestris* acetolactate decarboxylase (ALD6)**  ATGGCGCATAACGAGGTCTTTCAATATAGCATTATTAGCGCGCTTATGGATGGCGTCGCGTCTCACGGAACGCCGATTGCGAGAGTGTTAGAACATGGAGATCATGGCCTTGGCACGTTTCGTAATATGGTGGGCGAAATGATTGTTCTTGATGGCCAAGTCTATCAGATGAAAGCGGATGGCTCGGTTGTGCATATTGCCGACCCAGAAGCGACGGTCACACCGTTTGCGACAGTAACACGCTTTAGACCTACGACGGCGACGCGGGCGACAGTAGTGGGAAAAGCGGGAGTCGAAGCGTTACTTACAGGACTTTTTCCTCAAGCGCGCAATCATTTTATTGCGGTCCGTATGGATGGAGTGTTTCGTACAGTCAATGTCCGGACGGCGGGAGGACAACTTCGTCCTAGAGAAGGCATGGTAGATGTCTGTGCGAGACAAACAACACATACGTTTGAAGGTGTTAAAGGCACGATTGTAGGCTTTCGCTGCCCTGAATATGTGATGGGCATTAATGTCGCGGGCGATCATTTTCATTTTATTTCCGAAGATCGCCAACGGGGAGGCCATATTCTTGCGTTTGAAACGGAGGGCGATGTTGATATTGGAGCGGCGCAAATGAGCAAATTTCATCTTGAATTACCTACAGAAGATGAAGAGTTTAACGAGGCGACGCTTAAACTTCAAGCGCAACGTATTAAAGCGGTCGAAGGCTAA |

**References**

Bae, S.J., Kim, S., Hahn, J.S., 2016. Efficient production of acetoin in *Saccharomyces cerevisiae* by disruption of 2,3-butanediol dehydrogenase and expression of NADH oxidase. *Sci. Rep*. 6. <https://doi.org/10.1038/SREP27667>

Biswas, R., Yamaoka, M., Nakayama, H., Kondo, T., Yoshida, K.I., Bisaria, V.S., Kondo, A., 2012. Enhanced production of 2,3-butanediol by engineered *Bacillus subtilis*. *Appl. Microbiol. Biotechnol*. 94, 651–658. <https://doi.org/10.1007/s00253-011-3774-5>

Cho, S., Kim, T., Woo, H.M., Lee, J., Kim, Y., Um, Y., 2015. Enhanced 2,3-Butanediol Production by Optimizing Fermentation Conditions and Engineering *Klebsiella oxytoca* M1 through Overexpression of Acetoin Reductase. *PLoS One* 10, e0138109. <https://doi.org/10.1371/journal.pone.0138109>

Cripps, R.E., Eley, K., Leak, D.J., Rudd, B., Taylor, M., Todd, M., Boakes, S., Martin, S., Atkinson, T., 2009. Metabolic engineering of *Geobacillus thermoglucosidasius* for high yield ethanol production. Metab. Eng. 11, 398–408. <https://doi.org/10.1016/j.ymben.2009.08.005>

Erian, A.M., Gibisch, M., Pflügl, S., 2018. Engineered *E. coli* W enables efficient 2,3-butanediol production from glucose and sugar beet molasses using defined minimal medium as economic basis. *Microb. Cell Fact*. 17, 1–17. <https://doi.org/10.1186/s12934-018-1038-0>

Huang, S., Geng, A., 2020. High-copy genome integration of 2,3-butanediol biosynthesis pathway in *Saccharomyces cerevisiae* via in vivo DNA assembly and replicative CRISPR-Cas9 mediated delta integration. *J. Biotechnol*. 310, 13–20. <https://doi.org/10.1016/j.jbiotec.2020.01.014>

Jantama, K., Polyiam, P., Khunnonkwao, P., Chan, S., Sangproo, M., Khor, K., Jantama, S.S., Kanchanatawee, S., 2015. Efficient reduction of the formation of by-products and improvement of production yield of 2,3-butanediol by a combined deletion of alcohol dehydrogenase, acetate kinase-phosphotransacetylase, and lactate dehydrogenase genes in metabolically engineered Kl. *Metab. Eng*. 30, 16–26. <https://doi.org/10.1016/j.ymben.2015.04.004>

Ji, X.J., Huang, H., Zhu, J.G., Ren, L.J., Nie, Z.K., Du, J., Li, S., 2010. Engineering *Klebsiella oxytoca* for efficient 2, 3-butanediol production through insertional inactivation of acetaldehyde dehydrogenase gene. *Appl. Microbiol. Biotechnol*. 85, 1751–1758. <https://doi.org/10.1007/s00253-009-2222-2>

Kim, J.W., Kim, J., Seo, S.O., Kim, K.H., Jin, Y.S., Seo, J.H., 2016. Enhanced production of 2,3-Butanediol by engineered *Saccharomyces cerevisiae* through fine-tuning of Pyruvate decarboxylase and NADH oxidase activities. *Biotechnol. Biofuels* 9, 1–12. <https://doi.org/10.1186/s13068-016-0677-9>

Lau, M.S.H., Sheng, L., Zhang, Y., Minton, N.P., 2021. Development of a Suite of Tools for Genome Editing in *Parageobacillus thermoglucosidasius* and Their Use to Identify the Potential of a Native Plasmid in the Generation of Stable Engineered Strains. ACS Synth. Biol. 10, 1739–1749. <https://doi.org/10.1021/acssynbio.1c00138>

Nielsen, D.R., Yoon, S.H., Yuan, C.J., Prather, K.L.J., 2010. Metabolic engineering of acetoin and meso-2,3-butanediol biosynthesis in *E. coli*. *Biotechnol. J*. 5, 274–284. <https://doi.org/10.1002/biot.200900279>

Nilegaonkar, S., Bhosale, S.B., Kshirsagar, D.C., Kapadi, A.H., 1992. Production of 2,3-butanediol from glucose by *Bacillus licheniformis*. World *J. Microbiol. Biotechnol*. 8, 378–381. <https://doi.org/10.1007/BF01198748>

Radoš, D., Carvalho, A.L., Wieschalka, S., Neves, A.R., Blombach, B., Eikmanns, B.J., Santos, H., 2015. Engineering *Corynebacterium glutamicum* for the production of 2,3-butanediol. *Microb. Cell Fact*. 14, 171. <https://doi.org/10.1186/s12934-015-0362-x>

Schilling, C., Ciccone, R., Sieber, V., Schmid, J., 2020. Engineering of the 2,3-butanediol pathway of *Paenibacillus polymyxa* DSM 365*. Metab. Eng*. 61, 381–388. <https://doi.org/10.1016/j.ymben.2020.07.009>

Su, F., Xu, P., 2014. Genomic analysis of thermophilic *Bacillus coagulans* strains: Efficient producers for platform bio-chemicals. *Sci. Rep*. 4, 1–10. <https://doi.org/10.1038/srep03926>

Tong, Y.J., Ji, X.J., Shen, M.Q., Liu, L.G., Nie, Z.K., Huang, H., 2016. Constructing a synthetic constitutive metabolic pathway in *Escherichia coli* for (R, R)-2,3-butanediol production. *Appl. Microbiol. Biotechnol*. 100, 637–647. <https://doi.org/10.1007/s00253-015-7013-3>

Yang, T., Rao, Z., Zhang, X., Xu, M., Xu, Z., Yang, S.T., 2015. Enhanced 2,3-butanediol production from biodiesel-derived glycerol by engineering of cofactor regeneration and manipulating carbon flux in *Bacillus amyloliquefaciens*. *Microb. Cell Fact*. 14, 1–11. <https://doi.org/10.1186/S12934-015-0317-2/TABLES/3>

Yang, Z., Zhang, Z., 2018a. Recent advances on production of 2, 3-butanediol using engineered microbes. *Biotechnol. Adv*. <https://doi.org/10.1016/j.biotechadv.2018.03.019>

Zhang, L., Chen, S., Xie, H., Tian, Y., Hu, K., 2012. Efficient acetoin production by optimization of medium components and oxygen supply control using a newly isolated *Paenibacillus polymyxa* CS107. *J. Chem. Technol. Biotechnol*. 87, 1551–1557. <https://doi.org/10.1002/jctb.3791>

Zhang, X., Zhang, R., Bao, T., Rao, Z., Yang, T., Xu, M., Xu, Z., Li, H., Yang, S., 2014. The rebalanced pathway significantly enhances acetoin production by disruption of acetoin reductase gene and moderate-expression of a new water-forming NADH oxidase in *Bacillus subtilis*. *Metab. Eng*. 23, 34–41. <https://doi.org/10.1016/j.ymben.2014.02.002>

Zhang, X., Han, R., Bao, T., Zhao, X., Li, X., Zhu, M., Yang, T., Xu, M., Shao, M., Zhao, Y., Rao, Z., 2019. Synthetic engineering of *Corynebacterium crenatum* to selectively produce acetoin or 2,3-butanediol by one step bioconversion method. *Microb. Cell Fact*. 18, 1–12. <https://doi.org/10.1186/s12934-019-1183-0>
